# Curcumin: A Potential Weapon in the Prevention and Treatment of Head and Neck Cancer

**DOI:** 10.1021/acsptsci.4c00518

**Published:** 2024-10-14

**Authors:** Kateřina Veselá, Zdeněk Kejík, Michal Masařík, Petr Babula, Petr Dytrych, Pavel Martásek, Milan Jakubek

**Affiliations:** † BIOCEV, First Faculty of Medicine, Charles University, 252 50 Vestec, Czech Republic; ‡ Department of Paediatrics and Inherited Metabolic Disorders, First Faculty of Medicine, Charles University and General University Hospital in Prague, Ke Karlovu 455/2, 128 08 Prague 2, Czech Republic; § Department of Physiology, Faculty of Medicine, Masaryk University, Kamenice 5, 625 00 Brno, Czech Republic; ∥ Department of Pathological Physiology, Faculty of Medicine, Masaryk University, Kamenice 5, 625 00 Brno, Czech Republic; ⊥ First Department of Surgery-Department of Abdominal, Thoracic Surgery and Traumatology, First Faculty of Medicine, Charles University and General University Hospital, U Nemocnice 2, 121 08 Prague, Czech Republic

**Keywords:** Curcumin, Head and neck cancer, Tumor targeting, Metastasis, Nanoformulations

## Abstract

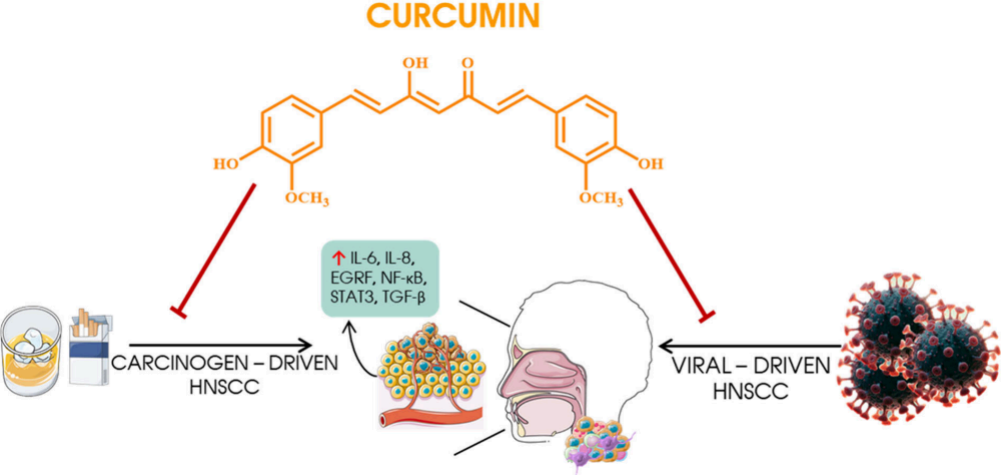

Head and neck cancers (HNC) are aggressive, difficult-to-treat tumors that can be caused by genetic factors but mainly by lifestyle or infection caused by the human papillomavirus. As the sixth most common malignancy, it presents a formidable therapeutic challenge with limited therapeutic modalities. Curcumin, a natural polyphenol, is appearing as a promising multitarget anticancer and antimetastatic agent. Numerous studies have shown that curcumin and its derivatives have the potential to affect signaling pathways (NF-κB, JAK/STAT, and EGFR) and molecular mechanisms that are crucial for the growth and migration of head and neck tumors. Furthermore, its ability to interact with the tumor microenvironment and trigger the immune system may significantly influence the organism’s immune response to the tumor. Combining curcumin with conventional therapies such as chemotherapy or radiotherapy may improve the efficacy of treatment and reduce the side effects of treatment, thereby increasing its therapeutic potential. This review is a comprehensive overview that discusses both the benefits and limitations of curcumin and its therapeutic effects in the context of tumor biology, with an emphasis on molecular mechanisms in the context of HNC. This review also includes possibilities to improve the limiting properties of curcumin both in terms of the development of new derivatives, formulations, or combinations with conventional therapies that have potential as a new type of therapy for the treatment of HNC and subsequent use in clinical practice.

Head and neck squamous cell carcinomas (HNSCC) cover a wide range of tumors from the nasal cavity, nasopharynx, oral cavity and lips to tumors of the salivary glands and larynx.^1−3^ Head and neck cancer (HNC) can be primarily caused either by tobacco or alcohol consumption or by human papillomavirus (HPV), especially HPV-16; a broader list of various head and neck tumors with an indication of localization is shown in Figure 1. Other risk factors for HNC include aging or other genetic factors. The incidence of HNC varies by region and correlates with exposure to tobacco-derived carcinogens and excessive alcohol consumption.^1,3−8^

**Figure 1:**
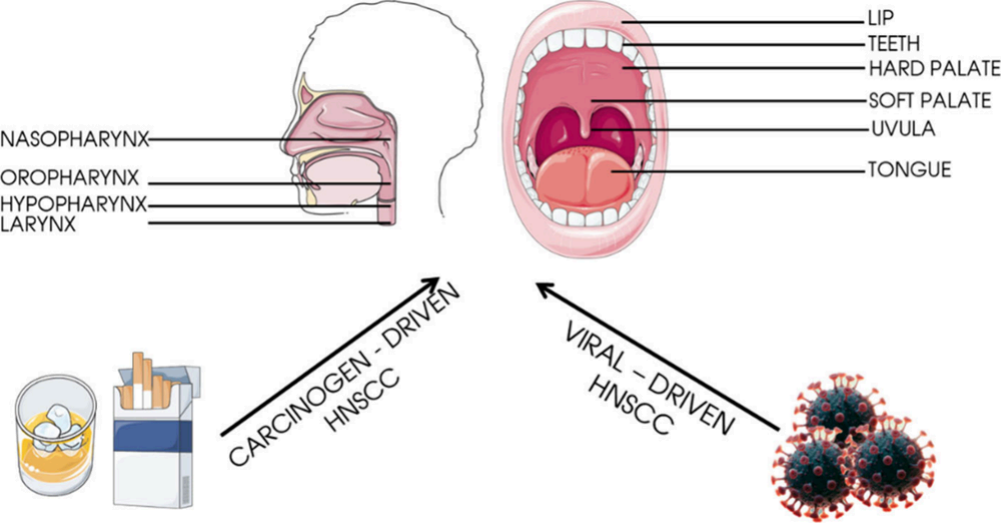
Locations of the most common head and neck tumors, such as the oropharynx, larynx, and nasopharynx. The appearance of head and neck tumors can be caused by carcinogens such as tobacco and alcohol or HPV.^2,3^ The figure was partly generated using ServierMedical Art, provided by Servier, licensed under a Creative Commons Attribution 3.0 unported license.

HNSCC is a difficult-to-treat group of cancers and is the sixth most common malignancy.^9^ Approximately 40% of patients are diagnosed at an early stage. Oral cavity tumors are usually asymptomatic and can present as persistent ulcerations. Nonoral types and sites of head and neck malignancy commonly have unclear symptoms and few obvious visible signs until late in the disease process, leading to delayed referral while treatment for other possible diagnoses is explored.^10^ Over the years, as can be seen in Figure 2, there has been a gradual increase in both patients diagnosed and deaths associated with HNC. It is known that head and neck tumors can potentially double in volume in 1–3 months, regardless of the size or site of origin. A delay in diagnosis and subsequent initiation of treatment thus significantly affects overall survival.^11^ Since the COVID-19 pandemic caused a delay in diagnosis, retrospective studies have shown that there has been an increase in patients with tumors classified as T3/T4.^11,12^

**Figure 2:**
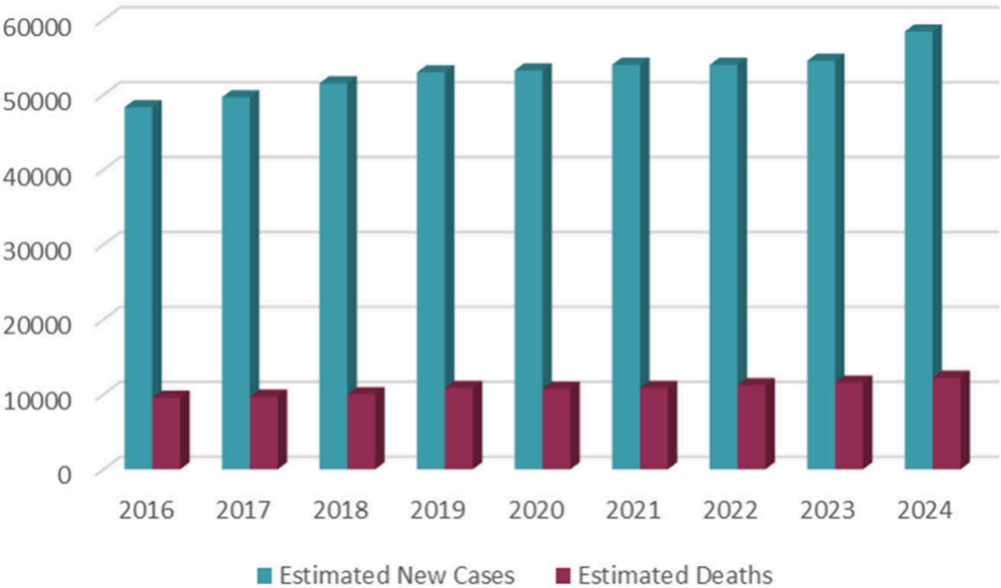
Monitoring the increase in new cases diagnosed and new deaths associated with HNC.^13−21^

The development of HNSCC is influenced by a number of genetic and epigenetic changes that lead to the inactivation of tumor suppressors and concomitant activation of proto-oncogenes. In the resulting tumor microenvironment, normal epithelial cells are transformed, and cells are propagated to form carcinoma. The most commonly affected pathway is p53, which activates the transcription of many genes affecting the cell cycle, apoptosis, DNA repair, or metabolism. In HNSCC, mutations in the TP53 gene often inactivate the p53 pathway, resulting in the accumulation of mutations and activation of oncogenes.^22−24^ The most commonly occurring p53 missense mutations (65–71% of HNSCC),^25,26^ in enhancement of proliferation and invasion, promote tumor relapses.^27^ In addition, mutant p53 in the HNSCC shows a higher incidence of cervical lymph node metastasis. These mutations then predispose HNSCC patients to drug treatment and radioresistance.

Late diagnosis is a cause of low survival rate, reported to be three to five months since diagnosis without any interventions.^2,7,28,29^ The treatment aims not only to improve survival outcomes but also to maintain organ function. HNC is usually treated with surgical resection followed by adjuvant therapy.^3,6^

Curcumin has promising potential as an anticancer and antimetastatic agent.^30−32^ Its anticancer activity has been demonstrated in various cancers including HNC. It has been shown to have inhibitory effects on cancer cells due to its antiproliferative and proapoptotic properties.^33−35^ The efficacy of curcumin on cancer cells is due to its multitarget mechanism. Known targets of curcumin include several transcription factors related to cell survival and proliferation (e.g., NF-κB (nuclear factor kappa-light-chain-enhancer of activated B cells), AP-1 (activator protein 1), STAT3 (signal transducer and activator of transcription 3), PPAR-γ (peroxisome proliferator-activated receptor gamma)), factors related to metastasis and angiogenesis (e.g., VEGF (vascular endothelial growth factor), ICAM-1 (intercellular adhesion molecule 1), COX-2 (cyclooxygenase-2), MMP-9 (matrix metallopeptidase 9)), receptors and kinases (e.g., EGFR (epidermal growth factor receptor) and ERK (extracellular signal-regulated kinase), JAK (Janus kinase), AKT (protein kinase B)), and cytokines (e.g., TNF-α (tumor necrosis factor alpha), IL-1β (interleukin-1 beta), IL-6 (interleukin-6) and MIP (macrophage inflammatory protein)).^34,36,37^

In the HNC pathogenesis, the disruption of NF-κB, JAK/STAT, and EGFR signaling pathways, particularly their intricate crosstalk, plays a pivotal role. This review meticulously explores not only the effects of curcumin on these pathways but also the possibility of enhancing the therapeutic potential of their inhibition within cancer biology by comparing the effects of new synthetic derivatives and new formulations of curcumin. Additionally, the clinical applicability of curcumin is rigorously assessed in this review.

## 1 Curcumin: From Chemical Structure to Biological Activity

**Figure 3:**
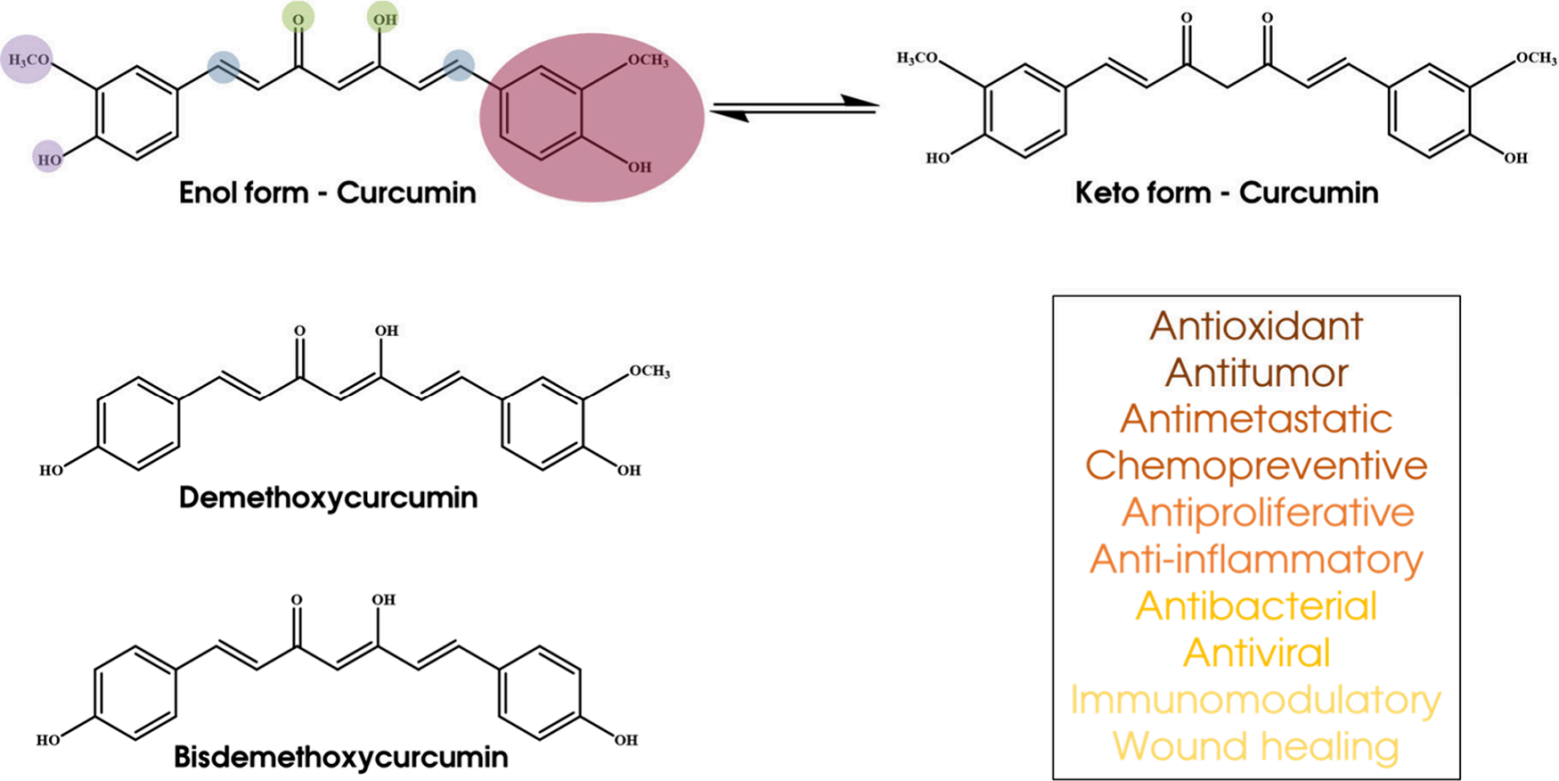
Structural formulas of curcumin and its derivatives with function groups (hydrogen bonding sites, Michael acceptor sites, and hydrophobic moieties) marked on the curcumin structure. Included are its anticancer properties.

Turmeric is a natural, yellow-colored polyphenol that is isolated from *Curcuma longa*. Curcumin, demethoxycurcumin, and bisdemethoxycurcumin are bioactive polyphenolic compounds contained in turmeric^33,36,38^ Turmeric has been used for centuries in China and India as a treatment for many illnesses such as inflammation and infection. The health effects of turmeric are attributed to an orange-yellow colored, lipophilic polyphenolic substance called “curcumin”, which is extracted from the rhizomes of the herb.^39,40^ The chemical structure of curcumin consists of two phenyl rings substituted with hydroxyl and methoxyl groups linked via seven carbon keto–enol linkers, as depicted in Figure 3. Curcumin has been shown to have many properties such as antioxidant, anti-inflammatory, antimicrobial, and anticancer.^41^

Curcumin has three main pharmacophores: an *o*-methoxyphenol group, an α, β-unsaturated β-diketone group, and seven carbon linkers, as can be seen in Figure 3. Accordingly, there are several important physicochemical properties associated with the biological activity and the effects of curcumin. For example, the *o*-methoxyphenol group and the methylene hydrogen are responsible for the antioxidant activity of curcumin, as these groups can donate electron/atom hydrogen to reactive oxygen species (ROS).^42,43^ Curcumin interacts with a variety of biomolecules through noncovalent and covalent bonding. The hydrogen bonding and hydrophobicity of curcumin arise from the aromatic functional residues. The keto–enol tautomerism of the central moiety, are responsible for the noncovalent interactions.^43−45^ Hu et al. performed a study using molecular docking to investigate interactions between curcumin and cancer therapeutic targets. They identified that curcumin has a strong affinity for AKT, TNF-α, STAT3, and EGFR.^46^

Curcumin has been shown to be safe up to high concentrations. In a clinical phase I study with 25 patients with high-risk or premalignant lesions, patients received ascending doses of curcumin with initial dose of 500 mg/day and maximal dose of 12,000 mg/day for 3 months. No treatment-related toxicity was observed up to a dose of 8000 mg/day; however, doses exceeding this threshold were impractical for oral administration due to the large volume required. Subsequent pharmacokinetics showed the peak of curcumin in the serum within 1 to 2 h after administration with a gradual decrease over 12 h.^47^ Despite its low toxicity, the clinical application of native curcumin has been limited due to its poor bioavailability caused by low solubility, physicochemical instability, pharmacokinetics, and especially its rapid metabolism.^44^ Modifications using conjugated π bonds and the occurrence of ortho–methoxy groups improved the anticancer and anti-inflammatory properties of curcuminoids compared to curcumin. For instance, phenyl rings are crucial for inhibiting tumor growth, and the addition of hydrophobic substituents such as methyl groups has also been associated with increased anticancer activity.^48−50^

For example, the synthesized CA15 monocarbonyl curcumin analogue increased the structural stability. This analogue showed enhanced inhibition of cell proliferation against laryngeal HEp-2 tumor cells. This analog inhibited NF-κB signaling through inhibition of IkappaB kinase (IKK) phosphorylation and prevented the degradation of IKK that binds to NF-κB. Furthermore, this analog exhibited enhanced inhibition of cell migration.^51^ Another of the targets is, e.g., the effect on the cell cycle, where synthetic curcumin analogs GO-Z078 and FLLL32 caused cell cycle arrest during the transition from G2 to M phase.^52,53^ Furthermore, the analog HO-3867 arrested the cell cycle in the G1 phase^54^ and the analog PAC inhibited the growth of head and neck tumors by destabilizing the cell cycle through suppressing cyclin D1 expression and increasing TP53 gene expression.^55^

As described above, despite its limitations, curcumin shows promising effects on HNSCC. Similarly, well-designed derivatives can affect HNC proliferation and migration by multiple mechanisms. For example, the most commonly studied targets are the NF-κB signaling pathway or its effect on the cell cycle and its associated molecular targets. However, its multitarget effect involves a number of oncogenic signaling pathways.

## 2 Curcumin’s Effect on Oncogenic Signaling

**Figure 4:**
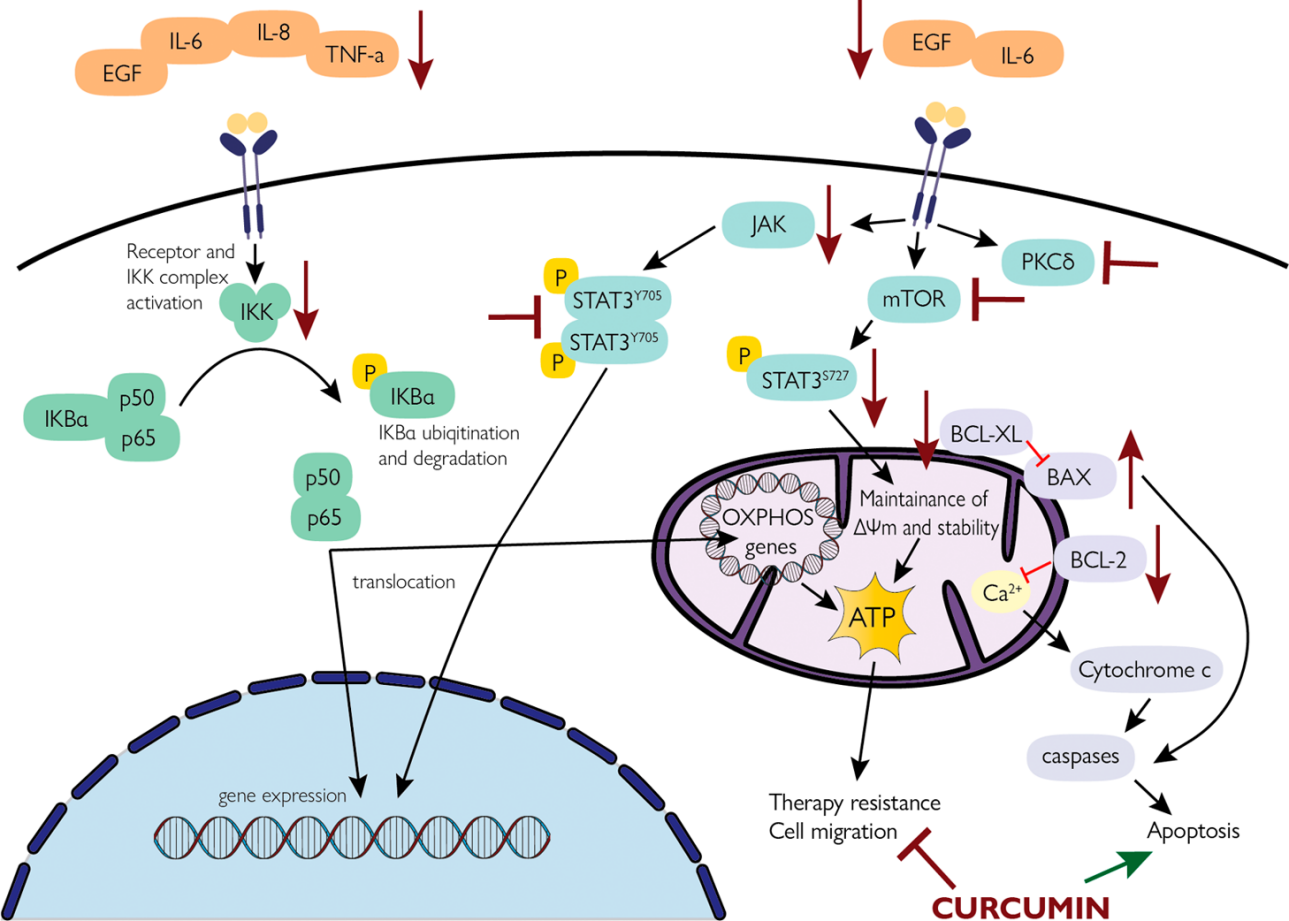
A simplified model of the curcumin effects on head and neck tumor cells.^59,74,76−78^ The EGFR pathway is complicated and multidimensional, leading to signal transduction cascades such as JAK/STAT. The PI3K/AKT/mTOR pathway is active in more than 90% of HNSCC cases, and mTOR is widely involved in cell transformation, proliferation, and survival. Upregulation of the JAK/STAT pathway is associated with cell proliferation, angiogenesis, and treatment resistance in HNC. Curcumin downregulates AKT and mTOR levels in HNSCC cells, as well as their phosphorylated forms. It also inhibits JAK2 expression and pSTAT3 production. Curcumin also downregulates NF-kB activation by inhibiting the IKKβ. This leads to the suppression of other genes involved in tumorigenesis via NF-kB such as COX-2, cyclin D1, MMP-9, IL-6 or IL-8. IL-6, interleukin 6; EGF, epidermal growth factor; TNF-α, tumor necrosis factor α; IL-8, interleukin 8; JAK, Janus kinase; pSTAT3, phosphorylated signal transducers and activators of transcription 3; mTOR, mammalian target of rapamycin; IKK, IkappaB kinase; BCL-xL, B-cell lymphoma-extra large; BCL-2, B-cell lymphoma 2; BAX, apoptosis regulator; PKC-δ, Protein kinase C delta. The figure was partly generated using ServierMedical Art, provided by Servier, licensed under a Creative Commons Attribution 3.0 unported license.

Despite ongoing research and advances in treatment, clinical outcomes and overall survival rates for HNSCC have not improved significantly over the past few decades. As a result, research into potential alternative and less toxic therapies for HNC continues with the aim of achieving a more favorable clinical outcome while reducing treatment morbidity. Curcumin is one of the potential candidates in the treatment of HNC.^56−59^Figure 4 shows a simplified scheme of the effect of curcumin on targets that affect the development of HNC. Treatment options for HNSCC include surgery, platinum-based chemotherapy, and radiation, all potentially leading to tremendous morbidity in patients.^60,61^ It has been studied that mitochondria influence tumorigenesis and metastatic spread by regulating signaling pathways associated with cell proliferation, and differentiation, including regulation of redox status or cell death pathways.^62,63^ ROS have been found to activate the (Phosphoinositide 3-kinases) PI3K pathway and the JAK/STAT3 pathway and can stimulate MAPK phosphorylation and cyclin D1 expression.^64,65^ The multiple functions and flexibility of mitochondria allow cells to adapt to changes in the microenvironment, which may contribute to tumor progression and chemoresistance.^63,66^ Resistance of HNSCC to chemotherapy and radiotherapy has been linked to mutations in the TP53 gene.^67^ Notably, it has been reported that the carbonyl groups of curcumin can form adducts with the thionyl groups of deubiquitinases,^68^ which play a critical role in maintaining the stability of the p53 protein.^69^ The loss of deubiquitinase functionality can lead to the accumulation of cytotoxic aggregates of mutant p53.^68^ Conversely, these protein aggregates can stimulate the induction of molecular chaperones, such as Hsp90, which serve to protect cells against apoptotic stress.^70^ However, it has also been documented that curcuminoids exhibit a direct inhibitory effect on Hsp90.^71,72^ In alignment with the aforementioned studies, research conducted in a hamster model of oral squamous cell carcinoma (OSCC), induced by 7,12-dimethylbenz[*a*]anthracene, demonstrated that the antitumor effects of curcumin were associated with a reduction in the protein levels of mutant p53.^73^ Consequently, there is considerable interest in the development of adjuvant chemotherapies to augment currently available treatments that may reduce side effects and toxicity without compromising therapeutic efficacy.

In this section, we discuss *in vitro* and *in vivo* studies supporting curcumin therapeutic activity in HNC, as well as some challenges regarding the development of curcuminoids as adjuvant chemotherapeutic agents.

A later study investigated the effects of curcumin on the growth and progression of HNC confirms its multitarget mechanism. Besides decreasing cell viability, curcumin also inhibited the cell cycle in the G2/M phase, disorganizing the cytoskeleton and subsequently altering cell morphology. In addition, it was also observed that curcumin downregulated the PI3K/AKT/mTOR signaling pathway.^74^

The mechanism of the curcumin analog, BDMC-A, has been studied in laryngeal cancer.^75^ BDMC-A was found to inhibit PI3K and pAKT, which is associated with the upregulation of p53. The upregulation of p53 in this case is associated with the induction of (BCL-2-like protein 4) BAX, an inhibitor of B-cell lymphoma 2 (BCL-2), and the related downregulation of BCL-2. As a result of these regulation, the mitochondrial membrane is disrupted and cytochrome c translocated to the cytosol, where caspase-9-mediated apoptosomes are formed and subsequently apoptosis is induced.^75^

Curcumin and its analogs have been found to downregulate the EGFR and thus affect other signaling pathways such as mTOR.^79^ The curcumin analogue FLLL12 showed the capability to affect EGFR and ATK at the mRNA level, thereby consequently inhibiting mTOR downregulation. In this study, FLLL12 was also shown to inhibit BCL-2 proteins, which repress mitochondria-mediated apoptosis.^80^ This is supported by a study in which curcumin was shown to inhibit HNSCC growth and enhance the effect of radiation both *in vitro* and *in vivo*. This effect is likely associated with downregulation of COX-2 and inhibition of EGFR phosphorylation.^35^ Should be also mentioned, that curcuminoids such as curcumin display direct inhibition activity against COX-2.^81^

Inhibition of the NF-κB pathway may lead to suppression of tumor growth, e.g., through inhibition of IKK, which is accompanied by decreased expression of stimulators of proliferation such as cyclin D1.^82^ The effects of curcumin on inhibition of the NF-κB pathway in HNSCC have been demonstrated both *in vitro* and *in vivo*, as can be seen in Table 1. For example, Lee et al. published, that natural derivative of curcumin DMC inhibits NF-κB phosphorylation and translocation to the nucleus in FaDu cells.^83^

**Table 1:** Curcumin *in Vivo* and *in Vitro* Effects on Head and Neck Tumors

*In Vivo*	Model	Effect
Curcumin + radiation	Athymic CD-1 nude mice (orthotopic implanted SCC-1 cells)	↓pEGFR; ↓COX-2; ↓tumor weight; ↓tumor size^35^
Curcumin analog FLLL12	Nude mouse xenograft model (subcutaneously injected Tu686 cells)	↓pEGFR; ↓EGFR; ↓pAKT; ↓AKT; ↓BCL-2^80^
Curcumin	SCID mice (subcutaneously injected SCC cell line SRB12-p9)	↓mTOR; ↓tumor growth^91^
Liposomal curcumin	Nude mice (subcutaneously injected CAL27 and UM-SCC1 cells)	↓NF-κB; ↓tumor growth^92^
Doxorubicin + curcumin-loaded peptide hydrogel (10 μM curcumin + 0.6 μM doxorubicin)	SCID mice (subcutaneously injected HSC-3 cells)	↑p53; ↑p21; ↑BAX; ↓tumor growth^93^
Curcumin	Xenograft mouse model (subcutaneously injected CAL27 cells)	↓NF-κB; ↓tumor growth^82^
Curcumin analog UBS109	Xenograft tumors in mice (subcutaneously injected Tu212 cells)	↓NF-κB; ↓tumor growth^94^
Curcumin analog EF31	Xenograft tumors in mice (subcutaneously injected Tu212 cells)	↓NF-κB; ↓tumor growth^95^

*In Vitro*	Cell line	Effect
Curcumin + radiation	SCC-1, SCC-9, KB	↓pEGFR; ↓COX-2^35^
Curcumin+ cisplatin	CAL27, UM-SCC1	↓NF-κB; ↓IKKβ^96^
Curcumin	SCC40	↓AKT/mTOR^97^
Curcumin	CCL23, CAL27, UM-SCC1, UM-SCC14A	↓IKKβ; ↓IL-6; ↓IL-8^98^
Liposomal curcumin	CAL27, UM-SCC1	↓NF-κB; ↓cyclin D1; ↓COX-2; ↓MMP-9; ↓BCL-2; ↓BCL-xL^92^
Curcumin	CCL23, CAL27, UM-SCC1	↓NF-κB; ↓pIκβ–α; ↓cyclin D1^82^
Curcumin	UM-SCC1	↓IFN-y; ↓IKKβ; ↓IL-10; ↓IL-2^99^
Curcumin	MDA 1986, Tu686, MDA 686LN, JMAR	↓pSTAT3; ↓IL-6^100^
Curcumin	FaDu, CAL27	↓NF-κB
Nanoformulation 5-FU and curcumin	SCC090, SCC152	↑p53; ↑p21; ↓Blc-2^101^
Curcuminoids	Detroit 562, HONE-1	↓NF-κB^102^
Curcumin	HHPC	↓NF-κB; ↓EGFR; ↓TNF-α; ↓STAT3; ↓IL-6; ↓BCL-2^103^
Curcumin	HEp-2	↓JAK2; ↓pSTAT3^104^
Curcumin	LSCC tissue, TU177, TU212, AMC-HN-8 and TU686	↓PI3K/AKT/mTOR^105^
Curcumin	AMC-HN-8	↓PI3K/AKT; ↓BCL-2^106^
Curcumin + irradiation	Hep-2, Hep-2-max	↓NF-κB; IKKγ; ↓cyclin D1; ↓BCL-2; ↓BCL-xL,^107^
Curcumin analog CA15	HEp-2	↓NF-κB; ↓pIKK;↓BCL-2; ↑BAX^51^
Curcumin analog BDMC-A	HEp-2	↓BCL-2; ↑BAX^37^
Curcumin	CAL27	↓BCL-2; ↑BAX^108^
Curcumin	SCC-9, FaDu	↓PI3K/AKT/mTOR^74^
Curcumin-MSNs	HN-5	↓BCL-2; ↑BAX^109^
Curcumin	HN-5	↓STAT3; ↓BCL-2, ↑BAX^110^

(p)EGFR, (phosphorylated) epidermal growth factor receptor; COX-2, cyclooxygenase-2; (p)AKT, (phosphorylated) protein kinase B; BCL-2, B-cell lymphoma 2; NF-κB, nuclear factor kappa B; IKKβ, inhibitor of nuclear factor kappa-B kinase subunit beta; IKKγ, inhibitor of nuclear factor kappa-B kinase subunit gamma; mTOR, mammalian target of rapamycin; IL-6, interleukin 6; IL-8, interleukin 8; IL-10, interleukin 10; IL-2, interleukin 2; MMP-9, matrix metallopeptidase 9; BCL-xL, B-cell lymphoma-extra large; IFN-y, interferon gamma; JAK2, Janus kinase 2; TNF-α, tumor necrosis factor; BAX, apoptosis regulator; (p)STAT3, (phosphorylated) signal transducers and activators of transcription 3; PI3K, phosphoinositide 3-kinases;. FaDu, pharynx squamous cell carcinoma; Tu686, laryngeal squamous cell carcinoma; CAL27, tongue squamous cell carcinoma; UM-SCC1, floor of mouth squamous cell carcinoma; HSC-3, tongue squamous cell carcinoma; Tu212, HNSCC; SCC-9, tongue squamous cell carcinoma; KB, human papillomavirus-related cervical adenocarcinoma; SCC40, esophageal squamous cell carcinoma; UM-SCC14A, floor of mouth squamous cell carcinoma; MDA 1986, oral cavity squamous cell carcinoma; MDA 686LN, oropharyngeal squamous cell carcinoma; JMAR, floor of mouth squamous cell carcinoma; SCC090, tongue squamous cell carcinoma; SCC152, hypopharyngeal squamous cell carcinoma; Detroit 562, pharyngeal squamous cell carcinoma; HONE-1, nasopharyngeal carcinoma; HHPC, human hypopharyngeal primary cells; HEp-2, larynx epidermoid carcinoma; LSCC, laryngeal squamous cell carcinoma; TU-177, laryngeal squamous cell carcinoma; AMC-HN-8, laryngeal squamous cell carcinoma; HN-5, tongue squamous cell carcinoma.

One of the main causes of cancer is a loss of balance between cell proliferation and cell death. When cells skip death due to the absence of apoptotic signals, uncontrolled cell proliferation occurs, leading to various types of cancer. Apoptotic signals are generated by two main pathways: the intrinsic pathway and the extrinsic pathway. The intrinsic pathway functions by stimulating the mitochondrial membrane to inhibit the expression of the antiapoptotic proteins BCL-2 and BCL-xL.^84−86^ Apoptosis plays a major role in maintaining the cell population. Curcumin disrupts the balance in the mitochondrial membrane potential, leading to an increased level of suppression of the BCL-xL protein. The extrinsic apoptotic pathway acts by increasing death receptors on cells and triggering (TNF)-related apoptosis.^84,87,88^ Derivatives and complexes of curcumin can also induce apoptosis in cancer cells and inhibit TNF action and production, which may provide multiple applications in cancer therapy.^89,90^

HPV-positive oropharyngeal carcinoma is characterized by degradation and inactivation of the tumor suppressor p53 and inactivation of the pRB pathway through upregulation of p16. The p53 pathway in HNSCC can be disrupted through multiple mechanisms, which may include hyperactivation of viral transforming genes (E6 and E7).^111,112^ Curcumin inhibits HPV16 E6/E7 transcription and restores the expression of tumor suppressor proteins p53, pRB, and PTPN13. In addition, curcumin suppresses the expression of HPV oncoproteins, and their expression is increased by the carcinogen tobacco smoke.^113,114^

The effects of curcumin on the HPV-16 positive oral carcinoma was studied in the 93VU147T cell line. Mishra et al. demonstrated that curcumin is not only a potent inhibitor of the activity of the host nuclear transcription factors AP-1 and NF-κB but also selectively suppresses the transcription of the HPV-16/E6 oncogene during the carcinogenic process in oral cancer cells.^88^

The synergistic effect of natural substances with conventional chemotherapeutic agents leads to an interaction that improves therapeutic results or reduces negative side effects.^115^ The curcumin analogue PAC enhanced the efficacy of cisplatin on oral cancer, where administration of 5 μM PAC reduced the IC_50_ of cisplatin 10×. Also, simultaneous use of PAC and cisplatin increased autophagy, ROS production and inhibition of cell migration through E-cadherin.^116^

Lindsay et al. investigated the synergistic effect of curcumin and metformin in HNSCC HPV-positive and HPV-negative cell lines.^117^ Curcumin and metformin showed antiproliferative and proapoptotic effects on all HPV^+^ (SCC-90, SCC-152) and HPV^–^ (SCC-6 and CAL27) cell lines. They also showed a probable synergistic effect on p53 expression (normal form) in the HPV^+^ lines. While treatment with curcumin or metformin alone showed a minimal decrease in p53 expression, the combined treatment showed a significant decrease in p53 expression. It should be noted, that in this case of HNSCC, it has been reported that the apoptotic effect of curcumin is associated with the activation of the normal form of p53.^93,95,118,119^ The observed results suggest that the cytotoxic effect of curcumin is not entirely dependent on p53.^117^ In HPV^+^ cell lines, the discrepancy in p53 expression after treatment may be due to the ability of the agent to regulate the expression of the viral oncoprotein. Curcumin has the ability to downregulate the HPV-E6 viral oncoprotein, which inhibits p53 function.^117^

### 2.1 Molecular Cross-Talk between the NF-κB and STAT3 Signaling

The pro-inflammatory cytokines IL-1β, IL-6, and interleukin 8 (IL-8) are mediators of the acute phase response, which is often associated with the induction of oxidative stress situations.^120−123^ Their multifactorial functions have been described in several organ systems and diseases. In addition to its role in the immune response, IL-6 has been reported to have metabolic functions.^124,125^ Sylvine Carrondo Cottin et al. observed that the secretion of anti-inflammatory cytokines in the blood is associated with the immune response seen in some patients with HNC prior to treatment. As well, they showed that daily cigarette consumption was a strong predictor of pro-inflammatory serum IL-6 levels in HNC patients.^126^

Furthermore, Jinno et al. found that the prevalence of cervical lymph node or distant metastasis was significantly higher in the group with high IL-6 expression (labeling index >30%). They also found that in the IL-6 overexpression group, IL-6 receptor (IL-6R) and activated/phosphorylated signal transducer and pSTAT3 were detected in almost all OSCC cancer cells, which indicates that STAT3 signaling is activated through stimulation of the autocrine loop between IL-6 and IL-6R.^127^

IL-8 is a cytokine of the pro-inflammatory chemokine family with a strong chemotactic capacity for neutrophils. IL-8 plays an important role in the inflammatory response present in the microenvironment in several cancers, including those of HNSCC, and is related to its ability to proliferate, invade, and metastatic spread. IL-8 expression correlates with poor prognosis in many cancers, including HNSCC.^123,128,129^

Cohen et al. studied the effect of curcumin on IL-6 and IL-8 production in HNSCC cell lines. They treated HNSCC cell lines CCL23, CAL27, UM-SCC1, and UM-SCC14A with increasing doses of curcumin and measured the IL-6 and IL-8 levels. Curcumin treatment resulted in the dose-dependent production suppression of IL-6 and IL-8 in all cell lines. In CCL23 cells, IL-6 production was reduced at curcumin concentrations of 25 μM, and in CAL27 cells, IL-6 inhibition was observed at curcumin concentrations higher than 100 μM. The concentrations of curcumin required for IL-6 inhibition for UM-SCC1 and UM-SCC14A cell lines were greater than 150 μM. The same trend was observed for IL-8 inhibition. All cell lines had similar levels of NF-κB; however, UM-SCC1 and UM-SCC14A had significantly higher levels of IKK and required significantly higher doses of curcumin before IL-6 and IL-8 inhibition occurred. Curcumin treatment resulted in inhibition of IKK activity and inhibition of IL-6 and IL-8 expression.^98^

#### 2.1.1 Nuclear Factor Kappa B

NF-κB is a family of transcription factors that includes RelA (p65), RelB, c-Rel, NF-κB1 (p50/p105), and NF-κB2 (p52/p100). There are several mechanisms of NF-κB activation, which are usually termed canonical or noncanonical pathway. In the canonical pathway, IKK phosphorylates IKKα at the two N-terminal serines, initiating its ubiquitination and proteasomal degradation, leading to nuclear translocation of NF-κB complexes, predominantly p50/RelA and p50/c-Rel heterodimers. The noncanonical NF-κB pathway involves various signaling molecules, such as lymphotoxin α, and leads to activation of the p52/RelB dimer.^130,131^ Genes regulated by NF-κB include those that control apoptosis, cell adhesion, proliferation, innate and adaptive immune responses, inflammation, cellular stress response, and tissue remodeling.^132,133^ Major targets of activated NF-κB include chemokines such as angiogenic factors (IL-1α, IL-6, IL-8, and TNF-α), cell cycle progressors and regulators of apoptosis (BCL-xL, cyclin D1, TRAF1, and TRAF2), and metastatic factors (MMP-9). The NF-κB pathway is frequently activated with the development and progression of a number of aggressive cancers, including HNSCC.^59,134−136^ In healthy cells, NF-κB activation is a strictly regulated process. It can be activated only after appropriate stimulation and then upregulates transcription of its target genes. NF-kB is activated by many different stimulators, including anti-inflammatory cytokines such as TNF-α, EGF, bacteria, lipopolysaccharides, viruses, and physical and chemical stresses. Constitutive activation of NF-κB in HNSCC is caused by the activation of IKKα. Expression of constitutively active NF-κB in HNSCC is mediated by the TNF signaling pathway.^59,135^ Elevated NF-κB in cancer cells is associated with high proliferation, angiogenesis, metastasis, antiapoptotic activity, and chemoresistance in cancer cells.

HNSCC express constitutive NF-κB activation, resulting in a global increase in proangiogenic cytokines such as IL-6 and IL-8.^137^ Chronic exposure to cigarette smoke causes inflammation that contributes to the activation of NF-kB signaling and the development of HNSCC. Cigarette smoke contains polyaromatic hydrocarbons and ROS that damage DNA and induce the production of proinflammatory cytokines such as TNF-α, IL-6, IL-8 and IL-1 in respiratory epithelial cells.^138^ DNA damage and binding of TNF-α and IL-1β to their receptors stimulate IKK/NF-κB signaling, leading to increased transcription of pro-inflammatory cytokines such as IL-6 and pro-angiogenic cytokines such as IL-8 and VEGF. The pro-inflammatory cytokines NF-κB can promote tumor growth and metastasis, and serum levels have been shown to correlate with treatment response and survival. Cigarette smoke has been shown to cause phosphorylation and degradation of IKKα leading to activation of NF-κB signaling in HNSCC and other cancer cell lines.^134,139,140^

In HNSCC cell lines expressing constitutively active NF-κB and IKK, curcumin treatment inhibits NF-κB activity via abrogation of IKK.68 In addition, curcumin treatment strongly inhibits NF-κB-regulated target gene expression and cell proliferation, inducing apoptosis, G1 phase arrest,^141,142^ and DNA fragmentation.^141^ This result was confirmed by a study showing that curcumin reduced smokeless tobacco-induced NF-κB and COX-2 activity in oral premalignant and cancer cells.^82,141−143^

Curcumin inhibits phosphorylation of IKKα and its subsequent ubiquitination, leading to inhibition of NF-κB translocation into the cytoplasm and, thus, inhibition of NF-κB activation. The inhibitory effect of curcumin on IKK causes inhibition of IL-6 and IL-8, which was also tested in HNC lines.^92^ Consequently, IL-6-mediated phosphorylation of STAT3 is inhibited, thereby inhibiting cancer cell proliferation.^100^

Wang et al. used liposomal curcumin as a treatment for head and neck tumors *in vivo* (nude mice) and *in vitro* (CAL27, UM-SCC1). They found that liposomal curcumin suppressed NF-κB activation and the expression of cyclin D1, COX-2, MMP-9, and antiapoptotic proteins (BCL-2, BCL-xL, Mcl-1L, and Mcl-1S) was reduced, indicating an effect of curcumin on the NF-κB pathway. In an *in vivo* nude mouse xenoimplant model, tumor growth was suppressed by liposomal curcumin. The mean tumor weight of untreated mice was 118 mg, whereas the mean tumor weight of mice treated with liposomal curcumin was more than 3 times smaller at 33 mg.^92^ The use of curcumin paste was also studied *in vitro* and *in vivo*, where it was confirmed that the nuclear expression of NF-κB was reduced and the tumor size was approximately halved.^82^

#### 2.1.2 Proteins of the Signal Transducers and Activators of Transcription (STAT) Family

Proteins of the STAT family mediate cellular responses to cytokines, such as IL-6, and growth factors.^144^ STAT proteins belong to the cytoplasmic transcription factor family and have seven members including STAT1, STAT2, STAT3, STAT4, STAT5A, STAT5B, and STAT6. Of all the members, STAT3 and STAT1 have received much attention and are also overexpressed in HNC.^145,146^ Dysregulation of STAT signaling is implicated in tumorigenesis and progression.^147^

STAT3 can be found in two isoforms: STAT3α and STAT3β. Both versions of STAT3β and STAT3α are transcriptionally active but show different functions under physiological and pathological conditions. Constitutive activation of STAT3α has an oncogenic role and plays a role in the activation of cell proliferation, maturation, and survival. In comparison, STAT3β is a potential tumor suppressor. The relative protein ratio of STAT3α: STAT3β is approximately 4:1.^148,149^ In the case of HNSCC, upon STAT3 activation, STAT3 is first translocated to the plasma membrane after the binding of cytokines (IL-6) or growth factors (EGF) to the respective cell surface receptors. Subsequently, STAT3 is activated by phosphorylation of a tyrosine residue in its Src homology 2 (SH2) domain (Tyr705) either directly by activated tyrosine kinase receptors or by intracellular nonreceptor tyrosine kinases. As a result of STAT3Y705 phosphorylation, spontaneous dimerization of the transcription factor is induced via a reciprocal phosphotyrosine-SH2 interaction between two STAT3 molecules, while constitutive activation of STAT3Y705 was found in HNSCC cell.^144^

Nuclear translocation of STAT3 was inhibited by curcumin. Inhibition of STAT3 activation by curcumin was reversible, although even 24 h after curcumin removal. In addition to inhibiting constitutive expression, curcumin also abolished IL-6-induced STAT3 activation in HNSCC cells.^100^ Curcumin inhibits IL-6 induced pSTAT3 and downregulates STAT3 signaling by suppressing JAK2 activation, which subsequently leads to inhibition of cell growth, colony formation, cell cycle arrest and subsequently apoptosis.^150,151^

Mitochondrial STAT3 requires pS727 to enhance Ras-dependent electron transport chain (ETC) activity and tumorigenesis. STAT3S727 may allow the incorporation of this protein into the inner mitochondrial membrane to promote oxidative phosphorylation and respiration via the mitochondrial ETC. This leads to increased ATP production, providing additional energy required for rapid oncogenic growth and cell division.^152^ Inhibition of STAT3S727 activation leads to delay or inhibition of IL-6-induced gene activation in response to cell signaling.^153,154^ Konduri et al. showed that curcumin reduced STAT3S727 phosphorylation in 769-p cells in a dose-dependent way. For a 20 μM concentration of curcumin, there was a reduction of about 31% and for a 50 μM concentration about 49%.^155^ It should be mentioned that in the case of HNSCC patients, significantly higher levels of both STAT3Y705 and STAT3S727 (*p* < 0.0001 and *p* < 0.004, respectively) were found in biopsy samples from tumor tissue with compare normal nonmalignant mucosa.^156^

The expression of cytokines, growth factors, and angiogenic factors is induced by STAT3, and the associated receptors in turn activate STAT3, creating a forward loop between the tumor and cells of the microenvironment.^157^ In HNSCC, IL-6 is associated with STAT3 activation by acting on the gp130 coreceptor in an autocrine/paracrine manner.^158^ Curcumin inhibits IL-6-mediated STAT3 phosphorylation and the expression of gp130 (called also IL-6R subunit beta), which is associated with this IL-6 dependent STAT3 activation.^159^ Curcumin also decreases the expression of JAK2 and inhibits the STAT3 phosphorylation, which leads to a decrease in the expression of genes that are regulated by STAT3, thereby increasing apoptosis of cancer cells.^104,144,151^

In HNSCC, aberrant activity of NF-κB and STAT3 is caused by growth factors and cytokines and promotes tumor cell survival and proliferation. For example, NF-κB and STAT3 are both activated by EGFR, and NF-κB induces IL-6 expression, which in turn also leads to STAT3 activation. Both proteins modulate the BAX/BCL-xL ratio in HNSCC. Targeting STAT3 leads to growth inhibition and increases apoptosis and down-modulation of BCL-xL expression.^160,161^

### 2.2 Curcumin and Its Ability to Influence Anticancer Activity through EGFR

**Figure 5:**
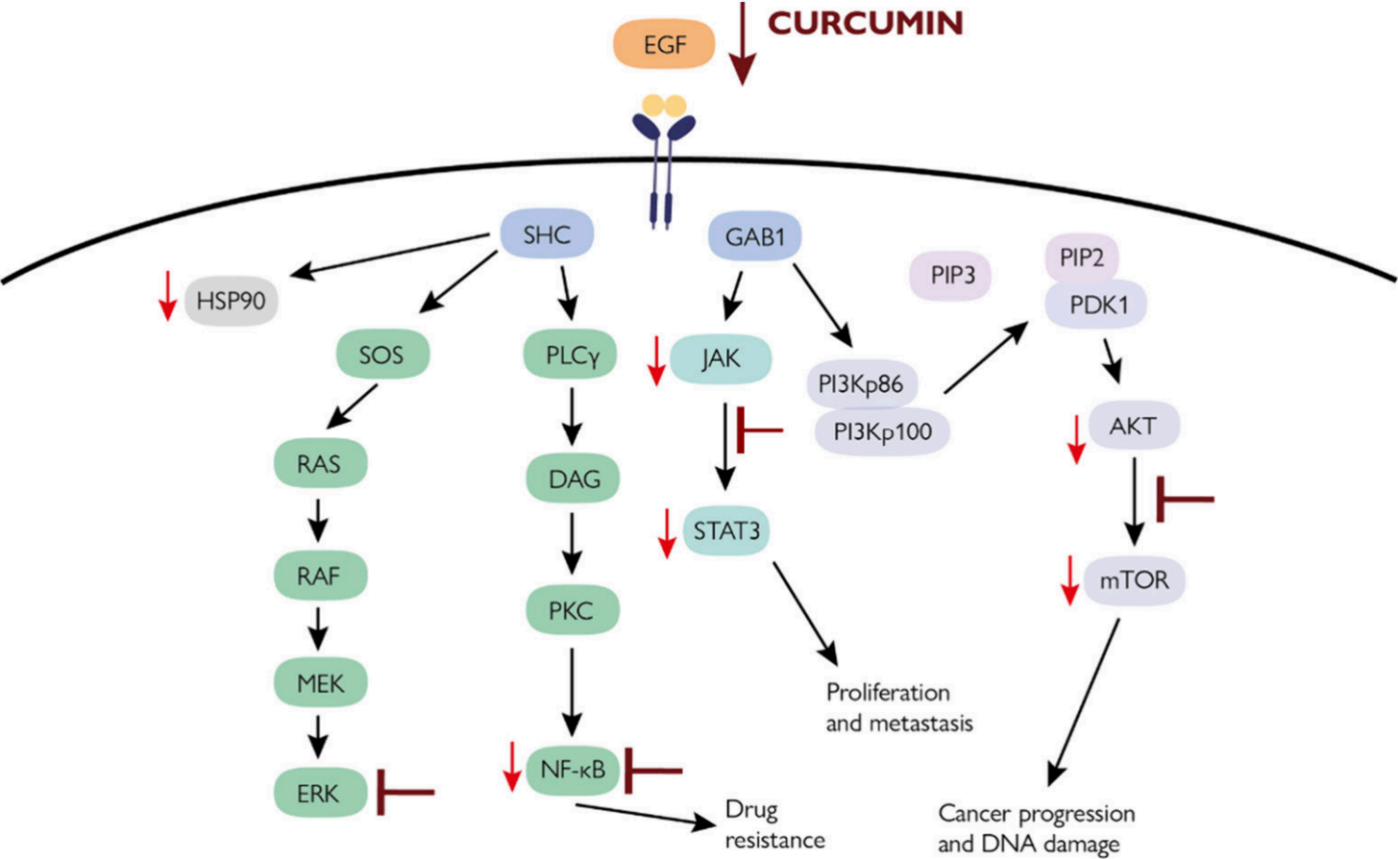
A simplified model of the effect of curcumin on the EGFR pathway.^76,79,171^ EGFR is overexpressed in almost 90% of the HNSCC cases. EGFR can be activated by ligands, such as EGF. EGFR activation leads to proliferation and intracellular signaling through cascades, such as the PI3K/AKT/mTOR pathway. Curcumin reduces the EGF-induced phosphorylation of EGFR. Furthermore, curcumin reduces the phosphorylation of AKT and STAT3. NF-κB plays a critical role in carcinogenesis, cell survival, chemoresistance and radioresistance, cancer cell invasion, and metastasis. EGF, epidermal growth factor; EGFR, epidermal growth factor receptor; PI3K, phosphoinositide 3-kinases; AKT, protein kinase B; mTOR, mammalian target of rapamycin; PIP2, phosphatidylinositol 4,5-bisphosphate; PIP3, phosphatidylinositol 3,4,5-trisphosphate; Gab1, GRB2 associated binding protein 1; SHC, SHC-transforming protein; PLCγ, Phospholipase C, gamma; DAG, diacylglycerol; PKC, Protein kinase C; NF-κB, Nuclear factor kappaB; RAS, class of protein called small GTPase; RAF, serine/threonine-specific protein kinases; MEK, mitogen-activated protein kinase kinase; ERK, “extracellular signal-regulated kinases; Hsp90, heat shock protein 90. The figure was partly generated using ServierMedical Art, provided by Servier, licensed under a Creative Commons Attribution 3.0 unported license.

The EGFR, known as ErbB1 or HER1, is a member of the transmembrane proteins; their ErbB family contains 4 transmembrane tyrosine kinases. Its stimulation by endogenous ligands, when EGF or TGF-α is bound, results in activation of intracellular tyrosine kinase and thus cell cycle progression.^162,163^ HER2, another member of the ErbB family is called the orphan receptor.^164,165^ HER2 crosstalk with other HER members such as EGFR, HER3 and HER4. The HER2:HER3 dimerization is one of the most potent oncogenic units that controls HER signaling.^166^ Curcumin downregulates EGFR signaling by inhibiting EGFR tyrosine phosphorylation.^167,168^ The EGFR pathway plays a critical role in the proliferation, migration, survival, angiogenesis, and invasion of cancer cells. High levels of EGFR expression correlate with poor prognosis and resistance to radiation therapy in various cancers, mostly HNSCC.^168,169^ EGFR is a major target for novel anticancer therapy in HNSCC, and other agents under development include small molecule tyrosine kinase inhibitors and antisense therapies, Figure 5.^167,170^

In addition to being expressed on the surface of healthy cells, EGFR is commonly expressed at high levels in various epithelial tumors, including HNSCC. The expression of EGFR in normal mucosa of HNC patients is 69-fold higher than that in healthy humans, and EGFR expression increases progressively according to the histological malignant transformation from hyperplasia to invasive carcinoma. Aberrant EGFR activation leads to increased proliferation and other tumor-promoting activities. Overexpression of EGFR is observed early in the carcinogenesis of HNSCC.^170,172,173^ Autocrine or paracrine activation by EGFR ligands is important for EGFR activation in HNC. Tobacco smoke, a classic contributor to HNC, can increase the production of amphiregulin and TGF-α, leading to direct activation of EGFR. Another pathway of EGFR stimulation is indirect activation of G-protein coupled receptors (GPCRs). GPCR ligands, such as prostaglandin E2 (PGE2) or gastrin-releasing peptide (GRP), are upregulated in HNC, and the subsequent activation of GPCRs leads to Src-mediated MMP activation; this causes cleavage and release of EGFR proligands (TGF-α, amphiregulin), ultimately leading to EGFR transactivation. Furthermore, following EGFR activation, expression of COX-2 and its downstream product PGE2 is increased, and PGE2 in turn transactivates EGFR, forming a positive feedback loop.^174−176^

Targeted therapy specifically targeting EGFR is an area of high interest in HNC research, because EGFR is potentially an integration point for convergent signaling. Despite recent advances in cancer diagnostics and anti-EGFR therapies, survival rates for patients with advanced HNC remain low due to resistance to anti-EGFR therapies.^177^ Promising preclinical studies have prompted the development of clinical trials testing EGFR inhibitors as monotherapy or in combination with conventional cytotoxic therapy with lower than expected response rates in advanced disease.^170^

Cetuximab (Erbitux), a monoclonal antibody (mAb) targeting the extracellular domain of EGFR, was approved for HNC either for advanced squamous cell carcinoma in combination with RT or as monotherapy for recurrent or metastatic squamous cell carcinoma progressing after platinum-based therapy.^178^ In a study by Chen et al., the synergistic effect of cetuximab with curcumin was studied in an *in vitro* model of cisplatin-resistant oral cancer. The combination of cetuximab with curcumin (20 μg/mL cetuximab and 40 μM curcumin) showed synergistic antiproliferative activity. While the viability of CAR cells after curcumin treatment was 48.5% and after cetuximab treatment was 85.8%, using the same concentrations, the synergistic effect showed a 38.6% viability. Furthermore, suppression of activated EGFR and MAPKs signaling was observed.^179^

Other FDA-approved treatments for patients with cancers that have EGFR mutations are tyrosine kinase inhibitors, such as Gefitinib. However, Gefitinib has shown only 10–15% clinical efficacy in patients with HNSCC.^178^ Hsiao et al. investigated the synergistic effect of gefitinib with curcuminoids (curcumin, DMC, and BDMC) on oral cancer. For example, a combination treatment of gefitinib (40 μM) with curcumin (25 μM) resulted in more than 2-fold reduction in cell viability compared to gefitinib and curcumin alone. Combinations of gefitinib with DMS or BDMC had a similar effect on the cell viability. In addition, an *in vivo* study was also performed, and treatment with gefitinib in combination with curcumin or DMC resulted in a reduction in tumor weight.^180^

Acquisition of resistance to therapy can be caused by various mechanisms, such as pre-existing mutations in EGFR or mutations acquired during therapy. In addition, tumors may develop resistance to treatment by acquiring mutations in domains in which therapeutic antibodies were originally intended to bind. Numerous mutations have been reported in the tyrosine kinase domain (TKD) and the ligand binding domain (ECD).^181,182^ HER2 is overexpressed and mutated in various types of cancer cells including HNSCC.^183^ Moreover, mutations occurring in HER2 cause resistance to chemotherapy drugs. First-generation EGFR-tyrosine kinase inhibitors (TKIs) such as gefitinib block EGFR autophosphorylation. Patients initially responding to EGFR-TKIs almost always develop drug resistance, which can arise from mutations in EGFR.^167,184^ Mutations in ECD EGFR are predominantly associated with cetuximab resistance in HNSCC. A difference in expression between primary tumor (gingival squamous cell carcinoma) and metastatic lesions was observed in a patient with long-term cetuximab treatment, who was found to be resistant. Metastatic lesions were found to have lower EGFR expression and higher E-cadherin expression with upregulation of epithelial-to-mesenchymal transition (EMT) genes.^185^ Curcumin, DMC and BDMC have high potential as HER2 inhibitors, overcoming their resistance gained by mutations. Indeed, curcumin in combination with gefitinib has been found to reduce EGFR activity and suppress receptor tyrosine kinases in gefitinib-resistant cells.^184^

Anisuzzaman et al. investigated the effects of a curcumin analogue *in vivo* and *in vitro* for the prevention and treatment of HNC and found that FLLL12 strongly inhibited the expression of and phosphorylation of EGFR and AKT. FLLL12 decreased the protein level of BCL-2 (antiapoptotic) and Bid (pro-apoptotic BCL-2 protein) and increases expression of apoptotic factor Bim. Nevertheless, full length protein form is inactive and is activated by caspase 8 through truncation, which inhibits expression of the full-length form. According to the proposed mechanism, less Bid expression strongly decreases the curcumin apoptotic effect.^80^

Curcumin inhibited ligand-stimulated EGFR activation suggests its potential to disrupt the autocrine loops formed in some advanced cancers. Blockade of EGFR can cause cancer cells to proceed to apoptosis. In addition, inhibition of EGFR abolishes the invasive potential of cancer cells. In HNSCC, there are mutations in the TKD of the EGFR gene.^181,186,187^ Most of these chemopreventive chemicals work by inhibiting other tyrosine kinases, such as c-Src, that are involved in linking activation of the G-protein coupled receptor to EGFR transactivation, which occurs extensively in established cancers. Curcumin enhances the antitumor activity of these agents (e.g., gefitinib) through inhibition of EGFR proliferation, phosphorylation, and induction of EGFR ubiquitination and apoptosis. These findings contemplate curcumin as an adjuvant to increase the spectrum of gefitinib use and overcome the ineffectiveness of gefitinib in patients.^188^

Curcumin has been shown to not only inhibit the tyrosine kinase activity of this receptor, but also deplete the protein itself by interfering with the function of the ATP-dependent chaperone protein GRP94, which is involved in maintaining the properly folded state of the receptor.^167,189^ Curcumin also target heat shock proteins (Hsp) such as Hsp90 by various mechanisms.^190^ Curcumin decreases its expression and dissociates the Hsp90 cochaperone p23, leading to inhibition of Hsp90 function. Hsp90 a molecular chaperone is involved in the maturation and stabilization of certain window gene receptor proteins that are essential for tumor growth, proliferation, and survival. A novel curcumin analogue, FM807, was studied and inhibited the growth of nasopharyngeal carcinoma. It was further found that binding of FM807 to the N-terminus of Hsp90 likely blocked the formation of Hsp90/receptor complexes, leading to the degradation of the Hsp90 receptor protein EGFR and inhibition of the downstream Raf/MEK/ERK and PI3K/AKT pathways.^191^

## 3 Curcumin in Tumor Targeting

**Figure 6:**
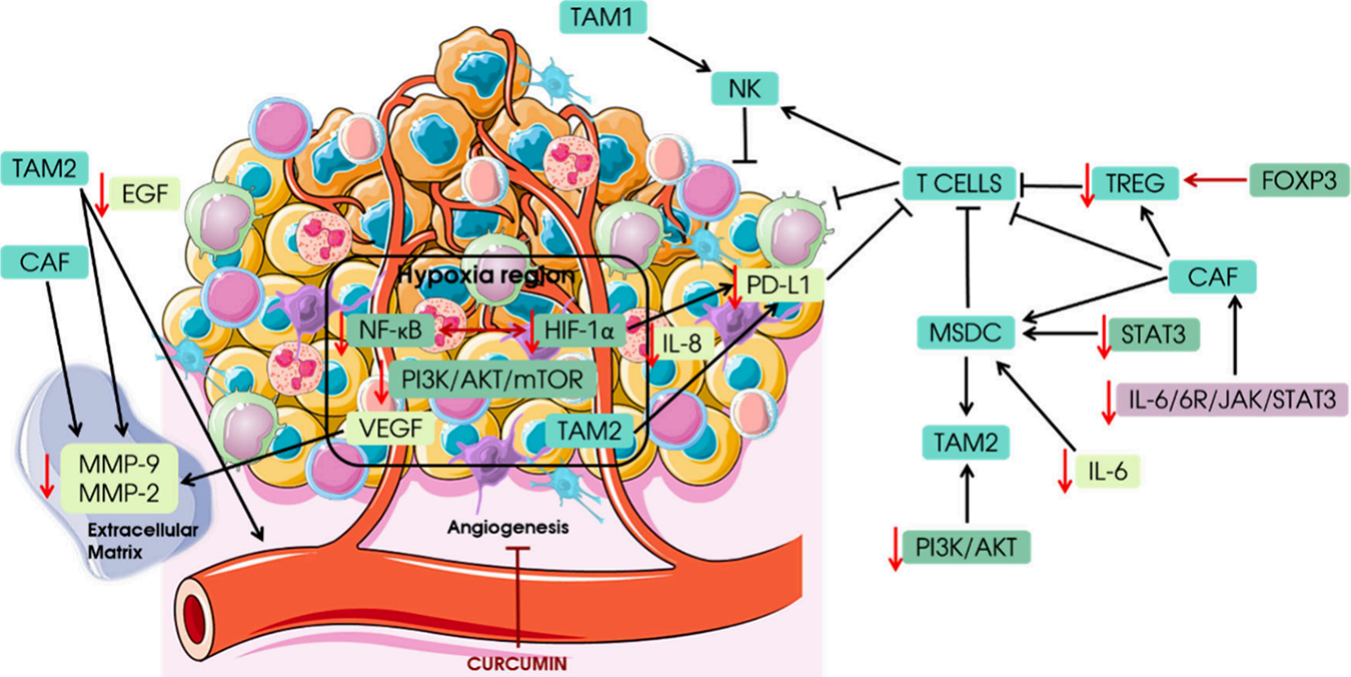
Schematic representation of the TME and the emergence of the CAF.^198−201^ Under normal conditions, fibroblasts are at rest. Differentiation and activation of fibroblasts to form CAFs are induced by different stimuli in the HNSCC microenvironment. HNSCC cells secrete a large number of cytokines and growth factors such as TGF-β, IL-1α, IL-1-β, IL-6, TNF-α and HGF. Thus, an important mechanism for CAF activation is the interaction between tumor cells and fibroblasts. HNSCC CAFs secrete cytokines and tumor-promoting factors necessary for inflammation, cell proliferation, tumor growth, invasion and metastasis, angiogenesis, CSC, and resistance to therapy. The main cell components of TME are TAM1/2, CAF, NK, TREG and MDSC. CAF, cancer associated fibroblast; EGF, epigermal growth factor; PI3K, phosphoinositide 3-kinases; AKT, protein kinase B; NF-κB, nuclear factor kappa B; P70S6K, Ribosomal protein S6 kinase beta-1; JAK, Janus kinase; STAT3, signal transducers and activators of transcription 3; IL-6, interleukin 6; IL-8, interleukin 8; VEGFA, Vascular endothelial growth factor; MMP-9, Matrix metallopeptidase 9; MMP-2, Matrix metallopeptidase 2; HIF-1α, Hypoxia-inducible factor 1-alpha; PD-L1, Programmed death-ligand 1; FOXP3, forkhead box P3; TAM1/2, Tubular aggregate myopathy 1/2; NK, Natural killer cell; MDSC, myeloid-derived suppressor cells. The figure was partly generated using ServierMedical Art, provided by Servier, licensed under a Creative Commons Attribution 3.0 unported license.

The tumor microenvironment (TME) of HNSCC consists of many different cell populations, such as cancer-associated fibroblasts (CAFs), infiltrating immune cells, and noncellular components of the extracellular matrix. CAFs are the predominant cell type in the tumor stroma, their main function in HNC is to maintain a favorable microenvironment for tumor cell growth and proliferation.^192^ Local normal fibroblasts (NFs) are the main source of CAFs in HNSCC, while tumor cells secrete growth factors, such as TGF-β and stromal cell-derived factor-1 (SDF1), facilitating the transformation of NFs into CAFs. TGF-β and fibroblast growth factor (FGF) cause opposing regulation of CAF effector genes, demonstrating that different activation approaches lead to different CAF phenotypes that have different effects on cancer growth. TGF-β is an activator of a phenotype called myofibroblastic CAF, which is characterized by upregulation of α-SMA.^193,194^ CAFs modulate the microenvironment primarily through the secretion of large amounts of autocrine and paracrine cytokines and other tumor-promoting factors critical for tumor cell proliferation, angiogenesis, invasion, inflammation, metastasis, and drug resistance. These factors include, for example, growth factors, cytokines, and chemokines, such as EGF, hepatocyte growth factor (HGF), and VEGF, as summarized in Figure 6. As tumor cells proliferate, they gradually consume oxygen and other nutrients, leading to tumor hypoxia, which is a hallmark of locally advanced HNSCC. By upregulating hypoxia-inducible factors (HIFs) such as VEGF, tumor cells overcome this problem.^195−197^ Overexpression of mediators of the hypoxic pathway, such as HIF-1α and HIF-1β, binds hypoxia response elements involved in tumor angiogenesis, and this effect may reduce the efficacy of radiotherapy and lead to tumor progression. Indeed, the hypoxic environment decreases ROS production, which reduces radiation-induced DNA damage and makes cells resistant to radiotherapy.^197^ Curcumin is known to suppress the protein synthesis of HIF-1α, a regulated subunit of HIF-1. Curcumin inhibits hypoxia-induced EMT-related genes mRNA expression and VEGF secretion.^167^

Curcumin was found to be able to reverse the CAF phenotype to that of peritumor fibroblast-like cells by downregulating the expression of α-SMA (α-smooth muscle actin) and inhibiting the secretion of pro-carcinogenic substances. Cytokines, including TGF-β, matrix metalloproteinases 2 (MMP-2) and SDF-1.^202^ When activated, CAFs express α-SMA, and α-SMA-positive myofibroblasts affect cancer progression and metastasis in HNSCC patients by producing extracellular matrix proteases and angiogenic/lymphogenic factors that induce EMT.^203,204^ In addition, curcumin led to the blockade of the CAF-mediated increase in CAL27 proliferation *in vitro* and *in vivo*. Ba et al. modulated CAFs in tongue squamous cell carcinoma by subcutaneously injecting CAL27 cells treated/untreated with curcumin into mice. Their results show that CAFs promote the tumorigenicity of CAL27 cells, and that curcumin attenuates the tumorigenicity of CAL27 cells by more than 30% by modulating CAFs.^202^

Kumar et al. investigated a novel curcumin analogue (H-4073) that exhibited significant inhibition of cell proliferation. In addition, the usage significantly reversed chemoresistance in cisplatin-resistant cell lines. Furthermore, it inhibited tumor angiogenesis by blocking VEGF production by tumor cells and directly inhibiting endothelial cell function. Overall, it inhibited JAK/STAT3, FAK, AKT and VEGF signaling pathways, which play important roles in cell proliferation, migration, survival and angiogenesis.^34^

### 3.1 Advanced Cancer: Metastasis and Drug Resistance

Metastasis is the primary cause of death for most cancer patients. Treatment of patients with metastatic disease in solid tumors remains predominantly palliative. The ineffectiveness of standard anticancer therapies against metastatic disease is reflected in the marked difference in survival from the time of diagnosis for localized and distant disease.^205,206^

The most common metastases in HNC are lymphatic metastases. Spread occurs progressively from the lymph nodes closest to the primary tumor to those furthest away. The incidence and spread of metastases in the HNC group ranges from 10 to 75% depending on the stage of the cancer. Stage T1 may show regional spread in 10–20% of cases, but metastasis occurs in 50–75% of cases for stages T3 to T4. The presence of a metastatic node has been reported to reduce 5-year survival by 50%.^206,207^ This difference is thought to be due to the greater disease burden in patients with metastatic disease and the intrinsic resistance of metastatic disease to most cancer therapies.

The resistance of metastases to conventional treatments is thought to be due to the numerous genetically unstable cell populations found in tumors.^208^ One of the key selection pressures shaping cancer cell evolution is the tumor microenvironment, which includes tumor cells, host stromal cells, the extracellular matrix, and immune system cells. One aspect of the interaction between the tumor and the microenvironment is mediated by the immune system through “immunoediting”. Immunoediting is proposed to be the process by which the immune system directs the selection of tumor cells toward an immune-resistant phenotype, including resistance to multiple host-secreted cytokines.^209,210^

STAT1 is a major transcription factor for IFNγ-related intracellular signaling and hence IFNγ-related tumor suppression. STAT1 is phosphorylated by JAK1/2 kinases at position Tyr701 and then translocates to the nucleus, where it binds to GAS (IFNγ-activated sequence) promoter elements, activating several hundred genes. These interferon-stimulated genes include the IFN/STAT1 signaling pathway. Therefore, the IFN/STAT1 pathway represents a signaling pathway that mediates crosstalk between components of the host microenvironment and tumor cells.^205,211^ In contrast, data show that in certain cellular contexts the IFN/STAT1 pathway can mediate cancer cell growth. This is supported by the study of Khodarev et al. where they found that STAT1 increased the radioresistance of transfected human HNSCC cells of the SCC-61 cell line.^212,213^ Curcumin is known to suppress STAT1 activation by directly inhibiting the phosphorylation of JAK1/2.^214,215^

Ruhul Amin et al. found that the FLLL12 analog of curcumin could be a potential new inhibitor for JAK2, whereby FLLL12-JAK2 interaction inhibits both IL-6-induced and constitutively active STAT3 phosphorylation in case of HNSCC.^216^ HNSCC, as well as other cancers, expresses programmed death ligand PD-L1. IFNγ and EGFR use JAK2 to transmit signals mediated by tumor cells. Concha-Benavente et al. investigated the mechanism by which these factors up-regulate PD-L1 expression in HNSCC cells. They found, among other things, that JAK2/STAT1 signaling is a common regulator of PD-L1 transcription, controlled by IFNγ and EGFR pathways. They further described that combined JAK2 inhibition and cetuximab-mediated EGFR blockade would lead to JAK2-mediated downregulation of PD-L1 in tumor cells, which would enhance the effector properties of PD-L1 and NK cells activated in the tumor microenvironment.^217^ Lin et al. reported that in HNSCC cell lines curcumin application decreases expression of PD-L1 and PD-L2. More importantly in the coculture CD8+ T cells and SNU1041 cells, curcumin stimulates immunotoxicity T cells. In the mouse model (SCC15 cell line) of HNSCC, expression of PD-L1, PD-L2 was significantly reduced after curcumin application. Similarly, tissues curcumin induced down-expression of PD-L1, PD-L2 protein was associated with restoration of antitumor immunity (mice model of tongue cancer).^218^

**Figure 7:**
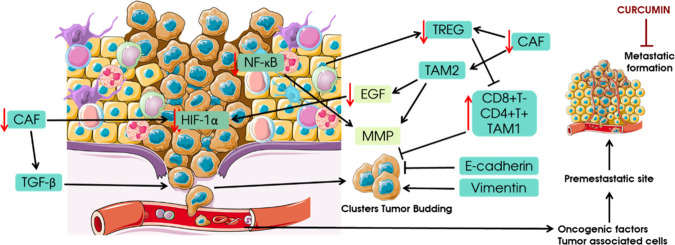
Schematic representation of possible metastasis development in HNSCC.^219−222^ Tumor dispersion, aggressiveness, and drug resistance occur after cancer cells undergo EMT, which is important for the development of metastasis. Inflammatory TME states can stimulate CD8+ T-lymphocyte responses to cancer cells. MMP-2 and MMP-9 are also known to be associated with lymph node metastasis and poor outcomes in laryngeal cancer. EGR, epigermal growth receptor; TGF-β, transforming growth factor beta; HIF-1α, hypoxia-inducible factor 1-alpha; NF-κB, nuclear factor kappa B; MMP, matrix metallopeptidase; EMT, epithelial-mesenchymal transition; CAF, cancer associated fibroblast. The figure was partly generated using ServierMedical Art, provided by Servier, licensed under a Creative Commons Attribution 3.0 unported license.

EMT is characterized by the loss of cell adhesion and upregulation of various extracellular matrix components, followed by increased migratory potential and enhanced invasiveness. EMT is associated with the loss of proteins involved in cell junctions, such as E-cadherin, and its loss is associated with tumor progression and increased metastasis in HNSCC patients, Figure 7.^192^

Curcumin appears to be a potential agent for the prevention of metastasis in oral cancer. Curcumin treatment not only decreased the expression of MMP-2 and MMP-9 to inhibit invasiveness in oral cancer, but also modulated the expression of EMT markers such as Snail, Twist and E-cadherin and induced the expression of p53, which is crucial for EMT repression.^223^

Metastasis and invasion promote the spread and growth of various cancer cells. Several studies have reported that curcumin exhibits its anticancer activity by suppressing the metastasis and invasion progression of cancer cell lines, which may be a promising therapeutic target for cancer treatment. The antimetastatic and anti-invasive mechanisms of curcumin are involved in the inhibition of transcription factors, inflammatory cytokines, proteases, protein kinases and regulation of miRNAs.^224,225^

There are many research studies that have shown that curcumin significantly inhibited metastasis in various types of cancer by regulating different signaling pathways. Potential antimetastatic mechanisms of curcumin include inhibition of transcription factors and their signaling pathways (e.g., NF-κB, AP-1 and STAT3), inflammatory cytokines (e.g., CXCL1, CXCL2, IL-6, IL-8), multiple proteases (e.g., uPA, MMPs), multiple protein kinases (e.g., MAPK, FAK), regulation of miRNAs (e.g., miR21, miR181b), and HIF-1α. For example, inhibition of NF-κB activation by curcumin leads to downregulation of expression of various proliferative genes and induction of apoptosis, thereby preventing invasion and metastasis of cancer cells. Furthermore, the level of activated STAT3 has been shown to be associated with metastasis in various types of cancers. Curcumin acts as a suppressor of the IL-6 cytokine signaling pathway and binds to the JAK activation loop, thereby blocking downstream signaling that requires phosphorylation and activation of STAT3.^225^

STAT3 is known to affect the expression of genes that promote migration and invasion. Curcumin significantly reduces the expression of MMP-2 involved in extracellular matrix degradation. Studies have shown that curcumin is a potent inhibitor of angiogenesis in HEp-2 cells *in vitro*. Curcumin appears to exert its antiangiogenic effect through inhibition of JAK2 expression and STAT3 phosphorylation.^104^

Mohankumar et al. investigated the mechanism role of bisdemethoxycurcumin analogue (BDMC-A) as a chemotherapeutic agent. They found that BDMC-A more effectively inhibited markers of invasion, angiogenesis, and metastasis compared to curcumin. Their results showed that BDMC-A inhibited the transcription factors NF-κB, p65, c-Jun, c-Fos, STAT3, STAT5, PPAR-γ, and β-catenin, which are responsible for tumor progression and malignancy. In addition, BDMC-A treatment decreased the levels of MMP-9, VEGF, TGF-β, IL-6 and IL-8 and increased the level of TIMP-2 (inhibitor of MMP).^226^

More than half of HNSCC patients experience resistance to therapies, including surgical resection, radiation therapy, and chemotherapy. Mechanisms that HNSCC cells use to avoid cell death after chemotherapy include DNA/RNA damage repair, inhibition of apoptosis, or activation of EGFR or NF-κB.^227,228^

Increased nuclear localization of NF-κB is associated with poor prognosis in patients with HNSCC. Moreover, NF-κB has also been reported to play a role in the development of resistance to chemotherapeutic agents in HNSCC. NF-κB has been implicated in HNSCC metastasis.^137^ Ming Yan et al. found that pharmacological blockade of NF-κB limited cell migration in highly metastatic Tb and TL HNSCC cell lines. Moreover, metastasis of TL cells in the lungs and lymph nodes in two animal models was severely suppressed by a selective NF-κB inhibitor, pyrrolidinedithiocarbamate.^229^

Thus, therapies designed to inhibit or block NF-κB activity would lead to downregulation of crucial cellular processes involved in tumor growth and the spread to metastatic sites. In addition, substantial research evidence has revealed that NF-κB plays an indispensable role in the development of both chemoresistance and radiation resistance in HNSCC, which has been identified as a primary cause of therapy failure.^230^

Curcumin has the ability to synergize the effects of the drug, thereby enhancing the global efficacy. Combination therapy using curcumin together with other anticancer drugs (5-fluorouracil, cisplatin, doxorubicin) has been described as a promising strategy to improve the therapeutic approach in the treatment of HNC. In addition, innovative systems (including nanoparticles, micelles, liposomes and hydrogel) for drug delivery could be adopted in the treatment of HNC. In addition, curcumin has been demonstrated as a chemical sensitizer for cancer cells and also as a chemo-protector for normal tissues at the molecular level.^86,231,232^

The combination of curcumin and cisplatin has been shown to result in enhanced growth suppression in HNSCC *in vivo* and *in vitro*. Curcumin inhibited IKKβ, leading to a reduction of NF-κB occupied sites in chromatin and a subsequent reduction in the level of NF-κB-mediated transcription. Thus, curcumin enhanced the therapeutic potential of cisplatin, which reduced NF-κB via the p53-mediated pathway. Due to this synergistic effect, lower, and therefore less toxic, doses of cisplatin can be used, which may suppress side effects.^96^

## 4 Curcumin Bioavailability: Limitation and Improvement

The potential health benefits of curcumin are limited by its poor solubility, low absorption from the intestine, rapid metabolism, and rapid systemic elimination. It is because of its limited water solubility, rapid metabolism, and excretion that its bioavailability is low. Orally administered curcumin at a dose of 500 mg/kg was shown to have a maximum serum concentration of only 0.06 μg/mL, indicating only 1% oral bioavailability. Therefore, efforts are being made to design and prepare new curcumin structures to identify analogs with more potent anticancer activities and better bioavailability, as can be seen in Table 3.^34,36,233,234^

In a study written by Pan et al., they investigated the pharmacokinetic properties of curcumin in mice injected intraperitoneally with 0.1 g/kg of curcumin. After 15 min, they detected 2.25 μg/mL curcumin in the plasma of the mice. One hour after administration, they subsequently determined curcumin levels in the intestine (177 μg/g), spleen (26 μg/g), liver (27 μg/g) and kidney (7.5 μg/g).^235^

When curcumin is administered orally, curcumin is rapidly metabolized and removed from the body by the formation of glucuronides and sulfates by conjugation in the intestine and may also interact with bile salts such that aggregation of bile salts dramatically changes the absorption and fluorescence parameters of curcumin. Blood concentrations of curcumin are extremely low after oral administration due to rapid metabolism in the intestinal wall and liver. When curcumin was administered orally at a dose of 10 or 12 g, the maximum plasma concentrations of curcumin in humans were still less than 160 nM.^234,236,237^

The chemical stability of curcumin depends on its molecular environment, with faster chemical degradation occurring under neutral or basic conditions than under acidic conditions. In aqueous media, the stability of curcumin is pH-dependent and this stability is commonly demonstrated by a change in the color of curcumin at different pH values. For example, at very low pH (<1), the color of curcumin in solution is red due to the predominance of its protonated form. In the pH range from 1 to 7, curcumin is yellow where its molecules are in the neutral form. At pH values > 7.5, curcumin takes on an orange color. The kinetic degradation of curcumin was studied in different buffer systems at different pH values. Curcumin is hydrolytically unstable at intestinal pH (pH gradually increases from pH 6 in the small intestine to approximately pH 7.4 in the terminal ileum), is rapidly metabolized, conjugated in the liver and excreted in the faeces.^238−240^ The results showed that at neutral pH, curcumin appears to be unstable when hydrolyzed into smaller products. In phosphate buffer at pH 7.4, curcumin was found to degrade within 30 min. As the pH increases, the rate of degradation of curcumin increases; for example, at pH 9 the rate of degradation is 10 times higher than at pH 8. The three main degradation products of curcumin are vanillin, ferulic acid and ferulic aldehyde (FA).^43,241−243^

FA has been found to have an inhibitory effect at the transcriptional level on the expression of some inflammatory mediators such as IL-6, TNF-α and iNOS, and an activating effect on the expression of some antioxidant molecules such as metallothioneins. Furthermore, FA reduced the translocation of NF-E2-related factor 2 (Nrf2) and NF-κB to the nucleus via decreasing the expression of phosphorylated IKK and subsequently inhibited IL-6 and NF-κB promoter activity in a luciferase assay.^244^

Han et al. investigated the effect of ferulic acid on an *in vitro* model of premalignant (SCC-83–01–82) and malignant (83–01–82CA) human oral epithelial cell lines. They found that ferulic acid treatment led to increased levels of cyclin B1 and cdc2 in both cell lines and p21waf1/cip1 was induced in the malignant cell line. Ferulic acid suppressed cell proliferation and caused G2 arrest in malignant oral cancer cells.^245,246^

As well as FA, vanillin has anti-inflammatory and anticancer properties. Liang et al. showed that vanillin suppresses MMP-9 transcription by inhibiting the NF-κB activity. They also found that it inhibits NF-κB activity through inhibition of phosphorylation and degradation of IKKα.^246,247^

The therapeutic effects of curcumin can be enhanced by synthesizing its derivatives with subsequent complexation of metal ions. The improved efficacy of curcumin derivatives may be due to better absorption and higher kinetic stability compared to native curcumin. A number of studies have investigated the antitumor activity of these derivatives in complex with metal ions.^9,142,248^

Effective delivery of curcumin using nanotechnology helps to address the limiting factors of curcumin such as solubility, *in vitro* and *in vivo* bioavailability, and pharmacokinetic properties such as rapid metabolism, degradation, and stability issues. By using various micro and nanoformulations, the drug can also be targeted to a specific site of action while minimizing potential toxicity to normal cells and tissues, leading to faster and more effective treatment, the advantages are summarized in Table 2 and also Table 3. Nanocarriers can improve the circulation time of the delivered therapeutic agent and can improve its accumulation at the pathological site using the so-called permeation and retention enhancement effect. By improving these clinically relevant parameters and enhancing its therapeutic potential, its ability to target cancerous tumors can be enhanced.^249−252^ Several strategies have been developed to produce nanocurcumin, each with its own set of advantages and unique properties. The effectiveness of a given nanomedicine depends on the manufacturing process and the carrier structure used for delivery. The use of a wide variety of nanostructures and nanocarriers such as micelles, hydrogels, liposomes, dendrimers is being studied.^253,254^

**Table 2:** Nanoformulation of Curcumin and Advantages of This Formulation Compared to Native Curcumin^a^

Formulation	Model	Advantages
β-Cyclodextrin-curcumin	C4–2, DU145	↑water solubility; ↑therapeutic efficacy^259^
Encapsulated PLGA nanoparticles	A2780CP, MDA-MB-231	↑water solubility; ↑inhibitory effect; ↑therapeutic efficacy^260^
Aqueous nanoparticulate formulation	PANC-1, MIAPaCa-2, K-562, MCF7, A549, HCT-116, BALB/c mice	↑water solubility; ↑stability; ↑bioavailability; ↑antiproliferative effect^261^
MPEG-P(CL-*co*-TMC) micelles	CT26, subcutaneous CT26 model	↑water solubility; apoptosis; ↓tumor growth^262^
Curcumin/LPPC	CT26, B16F10, female C57BL/6J mice (subcutaneously injected B16F10 cells)	↑apoptosis; ↓tumor growth; ↑cytotoxic activity^263^
Nanocurcumin loaded PLGA	DMH-induced male albino mice	↑permeability; ↑resistance to metabolic degradation; ↓inflammatory markers; ↓VEGF levels^264^

a C4–2, prostate cancer; DU145, prostate carcinoma; A2780CP, ovarian endometrioid adenocarcinoma; MDA-MB-231, breast adenocarcinoma; PANC-1, pancreatic ductal adenocarcinoma; MIA PaCa-2, pancreatic ductal adenocarcinoma; K-562, blast phase chronic myelogenous leukemia; MCF-7, invasive breast carcinoma of no special type; A549, lung adenocarcinoma; HCT-116, colorectal carcinoma; CT26, mouse colon adenocarcinoma; B16F10 murine melanoma cell.

**Table 3:** Formulations of Curcumin and Their *in Vivo* and *in Vitro* Effects on Head and Neck Tumors^a^

Formulation	Model/Subject	Effect
Curcumin liposomes	SCC9	↑bioavailability (inversely correlated to the size of the nanoparticles)^265^
Curcumin-loaded lipid-core nanocapsules coated with chitosan	SCC-25	↓Cell viability^266^
GZ17–6.02 (3 synthetic components; curcumin, harmine and isovanillin) [combination with cisplatin]	HN5, UM-SCC1 and OSC19, immortalized esophageal Het1A line from cancer free patient, glioblastoma line U87, and murine SCC/vII	↓*in vitro* assessments of HNSCC progression
		GZ17–6.02 = ↓EGFR; ↓AKT; ↓ERK1/2
		GZ17–6.02 + cisplatin = ↑antiproliferative effects^267^
Liposome-Encapsulated Curcumin	CAL27 and UM-SCC1 *in vitro* and nude mice xenograft (subcutaneously injected CAL27 and UM-SCC1 cells) *in vivo*	↓NF-κB; ↓cyclin D1; ↓COX-2; ↓MMP-9; ↓BCL-2; ↓BCL-xL; ↓Mcl-1L; ↓Mcl-1S; ↓tumor growth^92^
Peptide Hydrogel for the Local Co-delivery of Doxorubicin and Curcumin	HSC-3 and SCID mice (subcutaneously injected HSC-3 cells)	↑Apoptic response on HSC-3 cells; ↑antitumor efficacy; ↓tumor volume^93^
TriCurin (curcumin, epicatechin gallate and resveratrol)	UM-SCC47 and UPCI:SCC090 HPV-positive, preclinical animal model of HPV-positive HNSCC	↓HPV16E6; ↓HPV16E7; ↑p53; ↓tumor growth^119^
Microgranular curcumin	*n* = 18, newly diagnosed HNSCC of the oral cavity, oropharynx, hypopharynx or larynx	↓FGF-2 expression in postbiopsy samples; ↓FGF-2 serum levels; ↓GM-CSF; ↓IL-17^268^
Curcumin gum formulation	*n* = 10, clinical trial	↑Release of curcumin; ↑absorption of curcumin; ↑curcumin serum; ↓TNF-α; ↓CXCL1 (GRO-α)^269^

a EGFR, epigermal growth factor receptor; AKT, protein kinase B; ERK1/2, extracellular signal-regulated kinases 1/2; NF-κB, nuclear factor kappa B; COX-2, cyclooxygenase-2; MMP-9, matrix metallopeptidase 9; BCL-2, B-cell lymphoma 2; BCL-xL, B-cell lymphoma-extra large; Mcl, myeloid leukemia cell differentiation protein; FGF-2, fibroblast growth factor 2; IL-17, interleukin 17. SCC9, tongue squamous cell carcinoma; SCC-25, tongue squamous cell carcinoma; HN5, tongue squamous cell carcinoma; UM-SCC1, floor of mouth squamous cell carcinoma; OSC19, tongue squamous cell carcinoma; U87, glioblastoma; CAL27, tongue squamous cell carcinoma; HSC-3, tongue squamous cell carcinoma; UM-SCC47, tongue squamous cell carcinoma; UPCI: SCC090, tongue squamous cell carcinoma.

One of the promising methods of nanoformulation is the formation of liposomes.^30,255^ Liposomes are nontoxic, biocompatible, and biodegradable lipid vesicles that are increasingly utilized to encapsulate curcumin. In contrast, dendrimers provide sustained release and protection for curcumin. Liposomes not only protect curcumin from degradation but also prolong its duration of action and facilitate its delivery to the target site.^256,257^ Clinical trials have demonstrated that the application of liposomal formulations significantly increases curcumin levels in the blood.^30^ For instance, in a study involving healthy volunteers, the maximum and total curcuminoid concentrations in plasma were found to be 0.2 and 1.3 μg/mL, respectively, following the administration of Meriva (a curcuminoid phytosomal formulation containing 376 mg).^258^ In comparison, when a stand-alone curcumin extract (1.8 g) was administered, the maximum and total curcumin concentrations were only 15 and 203 ng/mL, respectively.

As shown in Table 2, nanoformulation of curcumin increases its water solubility, bioavailability and thus therapeutic efficacy. For example, Lin et al. encapsulated hydrophobic curcumin using a cationic liposome–PEG–PEI complex (LPPC) as a carrier. Thus, formulated curcumin exhibited 20 times greater cytotoxic activity against cells resistant to native curcumin. Furthermore, curcumin/LPPC also inhibited 60–90% tumor growth in mice bearing CT26 or B16F10 cells.

In addition to its use alongside anticancer drugs, curcumin can also be effectively combined with other natural polyphenolic compounds. Khalif et al. reported that the combination of epicatechin gallate and curcumin exhibits a strong synergistic effect against the proliferation and viability of both premalignant and malignant human oral epithelial cells.^270^ Similarly, Masuelli et al. found that the combination of curcumin and resveratrol was more effective in both *in vitro* and *in vivo* HNSCC models than both agents alone.^271^ In line with these findings, Piao et al. formulated TriCurin, a combination of curcumin, epicatechin gallate, and resveratrol in a specific stoichiometric ratio (4:1:12.5), which demonstrated potent antitumor activity against HPV^+^ HNSCC cell lines in both *in vitro* and *in vivo* studies.^119^ For example, in a preclinical animal model of HPV^+^ HNSCC, intratumoral injection of TriCurin significantly decreased tumor weight (by 85.5%, *p* < 0.01) and intratumoral levels of HPV16E6 (by 94.9, *p* < 0.001) compared to the vehicle group.

The biggest benefit of the formulation is the use of the full potential of curcumin to treat cancer, as is shown in Table 3. By appropriate formulation, the bioavailability of curcumin and its therapeutic efficacy can be increased, thereby increasing its ability to target cancer. All these parameters have been tested both *in vitro* and *in vivo*, as evidenced by the study of Wang et al., who prepared liposomal curcumin that suppressed the growth of head and neck tumors (CAL27 and UM-SCC1), further suppressed NF-κB activation, which subsequently reduced the expression of cyclin D1, COX-2, MMP-9, BCL-2, BCL-xL.^92^

### 4.1 Curcumin in Clinical Trials

Below in Table 4 is a summary of several clinical studies that examine the effect of curcumin in the treatment of cancer and the difficulties associated with treatment. Curcumin appears to be potentially useful in the treatment of cancer, possibly as an adjuvant in combination with other treatments.

**Table 4:** Curcumin, Its Derivatives, and Formulations in Clinical Trials

Agent/Formulation	Patient Characteristics	Effect
Curcumin gum formulation	Healthy adults	↓TNF-α; ↓CXCL1^269^
Curcumin	*n* = 39 (13 with dental caries, 21 with HNC, and 5 healthy volunteers)	↓IKKβ; ↓IL-8^99^
Nanomicelle curcumin	Prevention of radiotherapy-induced mucositis in HNC → 32 HNC patients	prevention of OM; ↓severity of OM^275^
Curcumin	Patients with CAS (anorexia-cachexia syndrome) in locally advanced or advanced HNC	↑muscle mass^276^
Basant polyherbal vaginal cream (containing extracts of curcumin) and curcumin vaginal capsules	HPV positive women without high grade cervical neoplasias, *n* = 287	↑HPV clearance rate in Basant; Curcumin caused ↑rate of clearance^277^
Curcumin	Oral leukoplakia, *n* = 223	good tolerance and durable clinical response^278^
APG-157, which contains turmeric extract	Oral cancer	↓IL-6; ↓IL-8; ↓IL-1β^272^
Curcuminoid capsule also contained piperine	Patients with colorectal cancer undergoing chemotherapy, *n* = 36	↓IL-1α; improve the ESR and serum levels of CRP in patients with stage 3 CRC^279^
Curcumin	Pancreatic cancer, *n* = 21	↓NF-κB; ↓STAT3; ↓COX-2^280^
Curcumin	Colorectal cancer, *n* = 126	↓TNF-α serum levels; ↑expression of p53 molecule in tumor tissue; ↑apoptotic tumor cells^281^

Kim et al. investigated the potential anti-inflammatory effect of curcumin in patients with HNSCC. Curcumin was found to suppress inflammatory cytokines such as IL-6, IL-8, granulocyte-macrophage colony-stimulating factor, and TNF-α, as well as IKKβ in the saliva of patients. They also suggested that IKKβ could be an acceptable biomarker for detecting the effect of curcumin in HNC because curcumin inhibited IKKβ activity in the saliva of HNSCC patients, and this effect was strongly correlated with reduced expression of a number of cytokines.^99^

Basak et al. conducted a double-blind, randomized, placebo-controlled phase 1 clinical trial with APG-157, a botanical drug containing multiple polyphenols including curcumin. They found that the treatment led to a reduction in the concentrations of IL-1β, IL-6 and IL-8 in the salivary supernatant of cancer patients. Furthermore, analysis of salivary microbial flora also showed a reduction in the number of *Bacteroidetes* species in the cancer subjects.^272^ The bacterial growth of *Bacteroidetes* in tumor tissue has been found in OSCC and early stage colorectal cancer.^273,274^

Curcumin can also be used as a prevention of oral mucositis (OM) and a supportive therapy in HNC patients requiring radiotherapy. Delavarian et al. investigated the effect of curcumin in the form of nanomicelles on OM in patients with HNC undergoing radiotherapy. They found that nanomicelle curcumin is an effective agent in preventing OM and reducing its severity. In the control group, all patients developed OM in the second week of radiotherapy. However, only 32% of the case group developed OM without obvious oral or systemic side effects.^275^

Curcumin as a natural substance shows excellent therapeutic effects *in vitro* and *in vivo*, either as a single therapeutic agent or as an adjuvant in combination with other drugs. In this perspective, clinical trials investigating the therapeutic potential of curcumin as a therapeutic agent for a number of diseases including HNSCC have been conducted or are still ongoing. This is evidenced by an ongoing Phase 2 clinical trial on HNSCC of the oral cavity and oropharynx using an adjuvant called APG-157 containing turmeric extract.^272^

## 5 Future

Curcumin has been shown to inhibit all three steps of carcinogenesis, initiation, promotion, and progression in an animal model of oral cancer. Curcumin has been shown to regulate the expression and activity of various molecules that play critical roles in cancer progression. As a single substance, curcumin can downregulate several molecular targets, including COX-2, HER2, EGFR, AKT, and VEGF, which are involved in critical cellular processes discussed earlier in this section and in signaling pathways. It has also been identified as an inhibitor of NF-κB and its target genes, and thus regulates several cellular processes: it inhibits cell growth and survival by suppressing the expression of BCL-2, cyclin D1, IL-6, COX-2, and MMP-9 proteins in HNSCC.^142,282−284^ As previously mentioned, one of the risk factors for HNSCC is tobacco smoke, and curcumin has the potential to mitigate the toxicity induced by smoking tobacco products.^285^ For instance, curcumin has been shown to repress ferroptosis in lung cells exposed to smoke.^286^ In this context, its effects on inflammatory NF-κB signaling should not be overlooked.^287^ Curcumin can also restore the expression of histone deacetylase 2, which represses the expression of pro-inflammatory genes such as IL-8 and macrophage inflammatory protein-2α.^288^ In accordance with the aforementioned findings, *in vivo* models have demonstrated that the application of curcumin leads to the alleviation of lung damage.^286,289^ The development of novel derivatives and formulations for the delivery of curcumin into biological systems presents a promising prospect not only for the treatment of primary and metastatic tumors but also for cancer prevention.

In the present time, many authors have reported that curcumin and other curcuminoids display potent antitumor and antimetastatic effect in the various types of cancer such as lung,^290^ breast,^291^ prostate^292^ or colorectal^293^ cancers. In the dependence of cancer type and experimental conditions, curcumin can directly repress growth of primary tumor, decrease migration of tumor cells, stimulate antitumor immune response, positively modulate gut microflora and target tumor microbiota.^30,294^ More importantly curcumin (including clinical trials) generally displays a positive effect on the usually used anticancer regiments.^295,296^

Current treatment regimens for HNSCC include the use of platinum-based chemotherapy. Cisplatin is not effective when used as a single agent, but its effectiveness is significantly increased when used with radiation therapy or in combination with other chemotherapy drugs. The mechanisms of these two agents through different growth signaling pathways suggest the potential for clinical use of subtherapeutic doses of cisplatin in combination with curcumin, allowing effective suppression of tumor growth while minimizing the toxic side effects of cisplatin.^96^ Platinum-based drugs such as cisplatin, oxaliplatin, and carboplatin have been used for many years for their therapeutic efficacy against a number of cancers (lung, liver, and ovarian). These drugs have been reported to have resistance issues and cause serious side effects such as renal toxicity, nausea and vomiting, leading to discontinuation of their use in clinical settings.^210,211^ Another possibility is that metal-curcumin complexes also increase the solubility, cellular uptake, and bioavailability, and enhance the antioxidant, anti-inflammatory, antimicrobial, and antiviral effects of curcumin.^297^ The metals interact with the ligand of curcumin, thereby altering the overall structure of curcumin and improving the biological efficacy of curcumin. The carbonyl group on the diketone moiety has been shown to be destabilized due to coordination of metal ions.^298,299^ In comparison with platinum based therapy, Ru-based drugs have significant advantages, including maximal therapeutic efficacy, availability in various oxidative states, and superior therapeutic efficacy in robust metastatic cancers.^210,216^ Ruthenium-curcumin complexes have been formulated and studied for their anticancer properties. Liposomal nanoparticles of the Ru-curcumin complex have been created and shown to effectively inhibit cell proliferation in HeLa cells.^300^ Li et al. compared the *in vitro* effects of the Ru-curcumin complex with cisplatin and curcumin alone. Their results show that their Ru-curcumin complexes have more than 4 times higher cytotoxicity for selected cancer cell lines than cisplatin or curcumin alone.^301^

Another option is the nanoformulation of curcumin, such as liposomal formulations. Curcumin can be incorporated into liposomes by various techniques and subsequently characterized by parameters -drug coating efficiency, size, *in vitro* release and *in vitro* cytotoxicity on squamous cell carcinoma cell lines. Liposomal curcumin was found to suppress the growth of HNSCC *in vitro* and *in vivo*. The studies suggest that liposomal curcumin could be utilized as a viable nontoxic therapeutic agent for HNSCC.^92,265^ A limitation of liposomal formulations is the method of liposome preparation. The preparation technique affects both the size of the resulting particle and the efficiency of encapsulation, as well as *in vitro* release and cytotoxicity. Gosangari et al. prepared 3 types of curcumin liposomes and found that the bigger particles have higher encapsulation efficiency. Using HNSCC-derived SSC-9 cells, they found that the liposomes with a size of about 0.157 μm had an IC_50_ at the concentration of 5 μM and for liposomes of about 0.077 μm the IC_50_ was 2.5 μM. Thus, it is necessary to select parameters that will result in efficient encapsulation and effective release of curcumin.^265^

Furthermore, the transmucosal administration of microgranular curcumin also leads to an increased bioavailability of curcumin more than double, which is associated with significant biological effects such as a significant reduction in FGF-2 expression. Already 1 h after administration of 3.6 g of microgranulated curcumin, the serum concentration of curcumin was approximately 77 ng/mL and the maximum concentration was measured to be approximately 404 ng/mL. Furthermore, IFNγ, IL-13, TNF-α and VEGF levels were measured at different time points (15 min, 30 min, 1 h, 2 h, 4 h and 3–4 weeks) and a decrease in the levels of these biomarkers was observed.^268^

In the meantime, new synthetic derivatives of curcumin can be developed that would exhibit better therapeutic effects and higher bioavailability. An *in vivo* study was performed where it was confirmed that long-term treatment with the synthetic curcumin derivative EF31 inhibited the phosphorylation of NF-κB p65 in mouse xenografts, which subsequently implied downregulation of cancer-promoting transcription factors such as angiogenesis and metastasis.^95^ Another example of a synthetic curcumin derivative with anticancer effects is UBS109, which suppresses tumor growth through the inhibition of NF-κB p65 phosphorylation. UBS109 has been shown to be effective in retarding the growth of Tu212 HNSCC tumors in mice and may be useful for the treatment of SCC head and neck tumors.^94^

## 6 Conclusion

Curcumin and its derivatives are promising substances with potential anticancer properties. Studies suggest that curcumin may have antimetastatic, antitumor, antioxidant, and anti-inflammatory effects that contribute to suppressing the growth and spread of cancer cells.^33,35,41^

Studies investigating the effect of curcumin on head and neck tumors suggest that curcumin may inhibit the growth and spread of these tumor cells, induce apoptosis and inhibit angiogenesis.^57,59^ In addition, curcumin suppresses inflammatory processes that may play a role in the development and progression of tumors. For example, as described in a study by Kim et al., who found that curcumin suppressed the inflammatory cytokines IL-6 and IL-8 in patients with HNSCC.^99^

Curcumin can influence several important signaling pathways that are associated with the development and progression of tumors. One of the major signaling pathways that curcumin affects is NF-κB, which is associated with inflammation, tumor growth and resistance to therapy.^132,133^ It has been shown that curcumin can inhibit NF-κB activation, thereby suppressing the growth and spread of cancer cells. In an *in vitro* study performed on HNC cell lines, curcumin was found to downregulate the NF-κB pathway and thus inhibit tumor cell proliferation.^100^

Another important signaling pathway affected by curcumin is the PI3K/AKT/mTOR pathway, which is associated with cell survival, growth, and migration. Inhibition of this pathway may also contribute to the suppression of cancer cell growth and spread.^97,105^

Furthermore, it has been found that curcumin can inhibit the migration and invasion of head and neck tumors and can influence several key processes associated with metastasis. This reduces the ability of cancer cells to penetrate into surrounding tissues and spread to distant sites in the body.^224,225^ Curcumin is also capable of affecting various pathways associated with cell migration, such as EMT, which is associated with increased migration of cancer cells.^192^ Curcumin modulates the expression of EMT markers (Snail, E-cadherin) and induces the expression of p53, which is important for EMT repression.^223^

The effect of curcumin in combination with conventional therapies has also been investigated and it has been found that curcumin can increase the sensitivity of cancer cells to these conventional therapies, e.g., chemotherapy and radiotherapy, suggesting that it could be used as an adjuvant therapy to improve existing treatments.^93,107^ The synergistic effect of curcumin and cisplatin was demonstrated, and their combination led to inhibition of IKKβ and enhanced growth suppression in HNSCC.^96^

Since curcumin is poorly soluble in aqueous solutions and thus has limited bioavailability, there is a need to find ways to maximize its therapeutic effects. There are a number of ways to increase the efficacy and utilization of native curcumin, either by using formulations—nanotechnology, liposomal formulations, micro- or nanoemulsions, or by preparing new synthetic derivatives.^36,233,234^ Studies suggest that curcumin derivatives may have stronger anticancer activity than that of curcumin itself. Curcumin derivatives are being designed to improve bioavailability, stability, and targeted activity. Properly designed derivatives may reduce toxicity and increase therapeutic potential in the treatment of HNC.^51,80,248^

The results obtained from *in vitro* and *in vivo* studies of curcuminoids suggest that curcumin and its derivatives may play an important role in the fight against HNC and may thus be a potential therapeutic target for future drug development. Future research should investigate the combination of curcumin with other treatments, such as chemotherapy and radiotherapy, to see whether these combinations can enhance the efficacy of HNC treatment. Another prospect is the development of curcumin derivatives and formulations that have improved pharmacological properties and targeted activity. New derivatives and formulations could provide new options for the treatment of HNC with a higher efficacy and reduced side effects.
